# Regional physicochemical filtering shapes microbiome–metabolome coupling at the Huangshui interface during Nongxiangxing Baijiu fermentation

**DOI:** 10.3389/fmicb.2026.1869375

**Published:** 2026-06-10

**Authors:** Wei Cheng, Na Li, Yingying Huang, Chunhui Wei, Ruilong Li, Yuan Zhang, Qiang Chang, Chao Jiang

**Affiliations:** 1 School of Biology and Food Engineering, Fuyang Normal University, Fuyang, China; 2 Brewing Science and Technology Key Laboratory of Sichuan Province, School of Food and Liquor Engineering, Sichuan University of Science & Engineering, Yibin, China; 3 Anhui Jinyu-Wan Distillery Industry Co., Ltd., Jieshou, China; 4 Anhui WenWang Brewery Co., Ltd., Linquan, China

**Keywords:** ecological filters, Huangshui, Nongxiangxing Baijiu, physicochemical-microbiome–metabolome interaction, regional variation

## Abstract

**Introduction:**

Huangshui (HS), the liquid fraction that accumulates at the bottom of fermentation pits, serves as a key ecological interface linking microbial diversity and metabolism between fermented grains and pit mud during fermentation. Although HS is increasingly recognized as a reservoir of microorganisms and flavor precursors, how regional heterogeneity shapes its microbiome–metabolome coupling remains unclear.

**Methods:**

HS samples derived from nine representative Nongxiangxing Baijiu (NXB) production regions were compared using physicochemical characterization, amplicon sequencing, volatile metabolomics, and ecological association analysis.

**Results:**

The results showed marked regional differences in acidity, nitrogen availability, mineral nutrient content, and organic acid composition, indicating distinct fermentation microenvironments. Lactic acid dominated the acid pool in all samples, whereas short- and medium-chain fatty acids varied substantially among regions, suggesting differences in carbon flux allocation and chain elongation activity. Bacterial communities were dominated by *Lactobacillus*, methanogenic archaea, and *Caproiciproducens*, whereas fungal communities were enriched with fermentative yeasts (*Pichia*, *Saccharomyces*, and *Kazachstania*). A total of 162 volatile organic compounds were identified, with esters as the predominant aroma class, showing clear regional differentiation. Furthermore, integrated correlation and network analyses indicated that regional physicochemical factors acted as ecological filters, shaping microbial guild assembly and volatile metabolite patterns.

**Discussion:**

*Lactobacillus* emerged as a central taxon associated with acid–ester balance, whereas methanogenic and chain-elongating taxa were linked to carbon redistribution and medium-chain fatty acid formation. Further, these findings support the revised view of HS as an active metabolic interface, highlighting its potential for origin discrimination and precise fermentation control in NXB production.

## 1 Introduction

Baijiu, a traditional distilled liquor originating in China, is produced via solid-state fermentation using *Jiuqu* as a saccharifying and fermentative starter. Its brewing process is characterized by “wheat for making Jiuqu, sorghum for brewing, mixed steaming and heating, and continuous mash fermentation,” forming a complex microecological fermentation system (Bai et al., 2024; Cheng et al., 2025a). Baijiu is the richest distilled spirit in terms of aroma (Chen L. et al., 2025), and Nongxiangxing Baijiu (NXB) is widely recognized for its rich ester aroma and strong regional characteristics among the major aroma types (Xu Y Q. et al., 2022; Cheng et al., 2026b). The quality of NXB is closely associated with its geographical origin, fermentation environment, microbial community structure, and technological parameters (Dong et al., 2024). Increasing evidence suggests that regional differences in fermentation ecology play a decisive role in shaping the flavor-formation patterns of Baijiu.

During fermentation, liquid exudates derived from starch and protein degradation accumulate at the bottom of fermentation pits. This viscous brown liquid, known as Huangshui (HS), originates from the downward migration of fermented grains (FG) that come in direct contact with pit mud (PM) (Cheng et al., 2025b; Cui et al., 2024; Zhang Y. L. et al., 2024). HS is enriched in diverse metabolites, including organic acids, alcohols, esters, and aldehydes, that arise from microbial metabolism, enzymatic hydrolysis, raw material transformation, and yeast autolysis (Zhang Y. L. et al., 2024). These metabolites serve as nutrients for microbial growth and act as precursors and active contributors to Baijiu flavor formation. Importantly, HS represents a transitional ecological interface connecting FG and PM, functioning as a microbial exchange medium and metabolite reservoir. Therefore, its microbial composition and metabolic output reflect the dynamic fermentation state of the pit system (Cheng et al., 2025b; Zhang Y. L. et al., 2024). Multiple factors influence the structure and function of HS microbiota, such as the production region, workshop age, climatic conditions, and brewing practices (Guo et al., 2024; Xu H. L. et al., 2022). Such environmental heterogeneity may reshape microbial community assembly and metabolic specialization, ultimately affecting the quality and aroma characteristics of NXB. Previous studies have primarily focused on the formation mechanisms, physicochemical properties, microbial diversity, and potential functional applications of HS (Gao et al., 2023; Guo et al., 2024). Further investigations have explored microbial succession patterns and identified bioactive components, including polysaccharides, which exhibit antioxidant and intestinal-protective functions (Cheng et al., 2025b; Xu H. L. et al., 2022).

However, systematic comparative studies that integrate the physicochemical properties, microbiome composition, and volatile metabolomic profiles of HS across multiple industrial production regions remain limited. In particular, the extent to which regional heterogeneity reshapes microbiome–metabolome interactions in HS and how these patterns correspond to previously reported community structures in FG and PM have not been fully elucidated. Although correlations between functional microorganisms and flavor compounds have previously been reported in Baijiu fermentation systems, the ecological associations and metabolic networks linking HS microbiota to flavor metabolite differentiation across different production regions remain unclear. This knowledge gap restricts the development of precise fermentation strategies and origin-traceability tools for NXB production.

Therefore, we hypothesized that regional physicochemical heterogeneity acts as an ecological filter that restructures microbial guilds in HS and redirects metabolite output toward distinct flavor trajectories. To test this hypothesis, we integrated physicochemical profiling, amplicon sequencing, volatile metabolomics, and ecological network analysis across HS samples derived from nine industrial NXB-producing regions.

## 2 Materials and methods

### 2.1 Sampling and pretreatment

**Figure 1:**
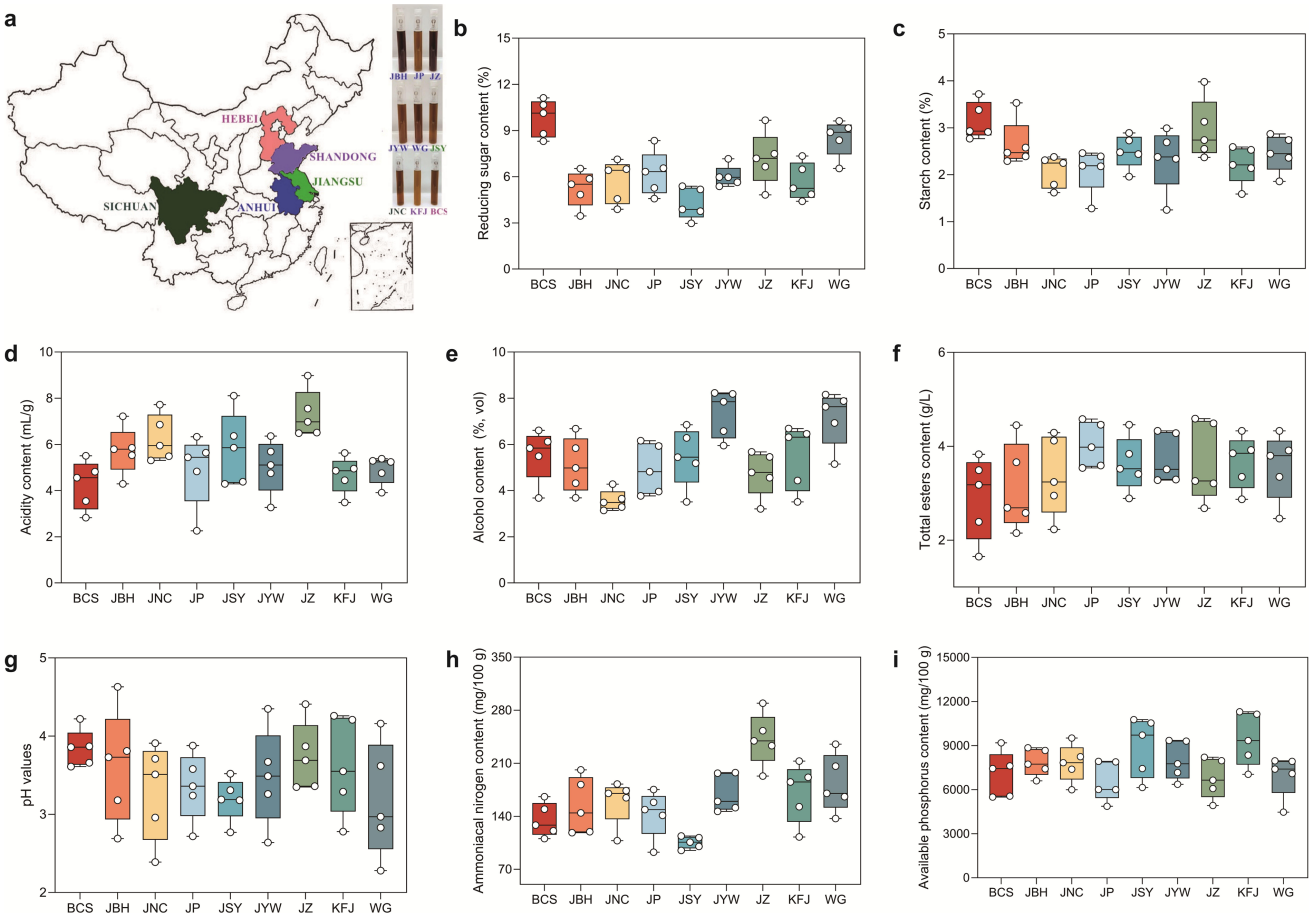
Geographic distribution and physicochemical characteristics of Huangshui (HS) samples obtained from different regions of China. **(a)** HS sampling locations across major Nongxiangxing Baijiu-producing regions in China. **(b)** Reducing sugar content, **(c)** Starch content, **(d)** Titratable acidity, **(e)** Alcohol content, **(f)** Total esters content, **(g)** pH values, **(h)** Ammoniacal nitrogen content, and **(i)** Available phosphorus content of the different HS samples.

Fresh HS samples were collected from nine industrial NXB distilleries located in five provinces in China (Figure 1a). The sampling sites were Qufu, Shandong Province (35.59°N, 116.99°E; KFJ); Chengde, Hebei Province (40.77°N, 118.18°E; BCS); Huai’an, Jiangsu Province (34.02°N, 119.20°E; JSY); Mianzhu, Sichuan Province (31.34°N, 104.18°E; JNC); and Fuyang (32.89°N, 115.81°E; JZ), Funan (32.70°N, 115.78°E; JP), Linquan (32.87°N, 115.19°E; WG), Jieshou (33.10°N, 115.27°E; JYW), and Bozhou (33.54°N, 115.47°E; JBH) in Anhui Province. The sample codes used for each HS sample are provided between parentheses.

HS was sampled immediately after completing pit fermentation. For each production region, three independent HS samples were collected from three different fermentation pits that had comparable pit ages (nearly 10 years of usage), adhered to similar production processes (following traditional NXB with fermentation for approximately 65 days), and shared similar operating parameters (sorghum used as the main raw material and FG with comparable starch and water contents). In addition, HS was sampled from different workshops that had nearly 10 years of usage in NXB brewing, demonstrated a suitable environment for NXB brewing, and exhibited differences in regional climate. Each sample (approximately 500 mL) was obtained by pooling the subsamples collected from five evenly distributed points at the bottom of the fermentation pit (four corners and the center) using a stainless-steel sampler. All samples were transported to the laboratory under refrigerated conditions and homogenized upon arrival.

For microbial analysis, equal aliquots from the three independent samples collected in each region were pooled to generate one region-level composite sample for DNA extraction, which was then stored at −80 °C until analysis. The remaining portions of the three individual samples were stored at −4 °C and used for physicochemical characterization and metabolite analysis. Before the physicochemical and flavor analyses, the HS samples were centrifuged at 10,000 × *g* for 15 min at 4 °C to remove insoluble residues, and the supernatants then collected and filtered for subsequent determination (Guo et al., 2024).

### 2.2 Physicochemical characterization and targeted metabolite quantification

The pH of each HS sample was measured using a calibrated pH meter (Mettler Toledo Instruments Co., Columbus, OH, USA). Alcohol, starch, and reducing sugar contents, as well as titratable acidity, were determined according to routine analytical methods established for HS derived from traditional solid-state Baijiu fermentation (Guo et al., 2024; China, 2012). Total acids and esters were quantified following the Chinese national standard method for Baijiu analysis (China, 2022). Ammonium nitrogen and available phosphorus levels were determined according to a standard analytical protocol used for PM evaluation (Zhang et al., 2020).

These physicochemical parameters were selected because they are commonly used to evaluate fermentation performance and are closely related to microbial metabolism and flavor formation in Baijiu systems. Major volatile compounds and principal organic acids were quantified via a gas chromatography (GC) system equipped with a flame ionization detector, according to standard analytical procedures (China, 2022). As lactic acid is the dominant nonvolatile organic acid present in HS, it was further quantified via high-performance liquid chromatography using an LC-16 system (Shimadzu, Kyoto, Japan) equipped with a Venusil MP C18 column (250 mm × 4.6 mm, 5 μm) and SPD-16 detector, following a previously reported method (Guo et al., 2024).

### 2.3 DNA extraction, PCR amplification, and amplicon sequencing

Microbial community analysis of the HS samples was performed according to previously reported procedures (Zhang et al., 2020). Total microbial DNA was extracted from each region-level composite HS sample using an E.Z.N.A. Soil DNA Kit (Omega Bio-Tek, Norcross, GA, USA), according to the manufacturer’s instructions. DNA quality and concentration were spectrophotometrically assessed, and DNA integrity verified using 1.0% (w/v) agarose gel electrophoresis.

For bacterial community analysis, the V3–V4 region of the 16S rRNA gene was amplified using primers 338F (5′-ACTCCTACGGGGAGGCAGCA-3′) and 806R (5′-GGACTACHVGGGTWTCTAAT-3′). For fungal community analysis, the internal transcribed spacer (ITS) region was amplified using primers ITS1F (5′-CTTGGTCATTTAGAGAGGAAGTAA-3′) and ITS2R (5′-GCTGCGTTCTTCATCGATGC-3′). PCR amplification, amplicon purification, and product quantification were performed as previously described (Xu et al., 2020). Sequencing libraries were prepared and sequenced on an Illumina NovaSeq 6,000 platform (Illumina, Inc., San Diego, CA, USA) by BioYigene Biotechnology Co., Ltd. (Wuhan, China).

Raw reads were quality-filtered using Trimmomatic (v.0.33), and primer sequences removed with Cutadapt (v.1.9.1). High-quality paired-end reads were merged using USEARCH (v.10) and subsequently length-filtered according to the expected amplicon size of each target region. Effective sequences were clustered into operational taxonomic units (OTUs) at 97% sequence similarity, and representative sequences taxonomically assigned against the Greengenes database (DeSantis et al., 2006; Edgar, 2010), with the aim of identifying 16S rRNA or ITS gene regions. Copy numbers were calculated according to the number of matched gene sequences for each taxon. Additionally, when multiple genome assemblies were available for a given taxon, selection was based on assembly quality and completeness. For taxa that could not be resolved at the species level, gene copy numbers were assigned using the average value at the lowest reliable taxonomic rank. Subsequent bioinformatics analyses were performed using QIIME2 and R software (v.3.2.0). Alpha diversity indices were calculated based on the OTU table, rarefaction curves generated to assess sequencing depth, and beta diversity evaluated using Bray–Curtis dissimilarity.

As the microbial DNA derived from the three pits in each region was pooled before sequencing, the sequencing results were interpreted as region-level composite profiles rather than as pit-level biological replicates. Accordingly, diversity comparisons and downstream association analyses were intended to capture broad regional patterns rather than within-region variance.

### 2.4 Untargeted volatile profiling via HS-SPME-GC–MS

Volatile organic compounds (VOCs) present in HS were analyzed using headspace solid-phase microextraction coupled with GC-mass spectrometry (HS-SPME-GC–MS), with minor modifications to a previously reported method (Gao et al., 2023). Briefly, 5.0 mL diluted HS solution (2.0 mL HS mixed with 3.0 mL ultrapure water) was transferred into a 20-mL headspace vial, to which 20 μL of an internal standard solution [2-octanol, as used by Gao et al. (2023); 0.0505 g/L] was subsequently added. The vial was equilibrated at 60 °C for 15 min under magnetic stirring. VOCs were extracted using DVB/CAR/PDMS fiber (50/30 μm; Supelco, Bellefonte, PA, USA) for 50 min at 60 °C with continuous agitation (300 rpm). After extraction, the fiber was immediately inserted into the injection port of the GC system and thermally desorbed at 250 °C for 5 min. VOC separation was performed on a GC–MS system (GC-8860, MS-5977B MSD; Agilent Technologies, Santa Clara, CA, USA) equipped with an HP-WAX capillary column (30.0 m × 0.25 mm × 0.25 μm; Agilent Technologies).

The GC–MS operating conditions used were the same as those described by Kang et al. (2022). Helium was used as the carrier gas, and the injector temperature set to 250 °C. The oven temperature program was as follows: 40 °C for 2 min, then increased to 120 °C at 5 °C/min and held for 3 min, and finally increased to 230 °C at 10 °C/min and held for 5 min. The ion source and interface temperatures were set at 230 °C. Compounds were tentatively identified by comparing their mass spectra with those in the NIST12 library, and only compounds with a similarity index >80% and retention behavior consistent with the C7–C40 range were retained (Cheng et al., 2026b). Relative quantification was performed using the internal standard method, and compound concentrations (mg/L) calculated from the ratio of the compound peak area to that of 2-octanol (Cheng et al., 2026b; Gao et al., 2023).

### 2.5 Statistical analysis

All physicochemical parameters and VOC data were expressed as mean ± standard deviation, and each determination performed in triplicate. Statistical analyses were conducted using R software (v.4.1.1). Differences among the HS samples obtained from different NXB production regions were evaluated using one-way analysis of variance, followed by the appropriate post-hoc multiple comparison tests. Differences were considered statistically significant at *p* < 0.05 and highly significant at *p* < 0.01.

Principal component analysis (PCA) was performed to visualize the overall variation in physicochemical and volatile metabolite profiles among the regional HS samples. To explore the potential associations among microbial communities, environmental variables, and flavor metabolites, Mantel tests were performed based on Bray–Curtis distance matrices. Spearman’s rank correlation coefficients were calculated to construct association networks between the dominant microbial genera and selected metabolites or physicochemical variables. Only statistically significant correlations were retained for network visualization, which was performed using Cytoscape (v.3.9.1) (Qiu et al., 2024).

## 3 Results and discussion

### 3.1 Regional heterogeneity defines distinct HS ecological niches

The pronounced regional variations observed in physicochemical properties of HS indicated that samples obtained from the nine NXB production regions represented distinct fermentation microenvironments rather than a uniform liquid by-product (Figures 1b-i, andFigure 2). Core variables related to carbon availability, acidity, alcohol level, and nutrient status differed substantially among the regions. The reducing sugar content ranged from 5.20–9.90% and that of starch from 2.03–3.23%, with BCS showing the highest residual carbohydrate levels and JSY and JNC showing comparatively lower values. Acidity varied from 4.72–6.43 mL/g, whereas the pH remained consistently low (3.35–3.76), confirming that HS constitutes a strongly acidic niche. Alcohol content also markedly differed among the regions, ranging from 4.39% vol in JZ to 7.32% vol in JYW. In parallel, ammonium nitrogen and available phosphorus levels displayed considerable variation, with JZ showing the highest ammonium nitrogen concentration and JYW showing the highest phosphorus level, whereas JSY consistently showed relatively low nutrient availability. Collectively, these differences suggest that HS derived from different production regions is characterized by distinct combinations of substrate supply, acid stress, and nutrient status, all of which are likely to act as ecological filters for microbial survival and metabolic activity.

**Figure 2:**
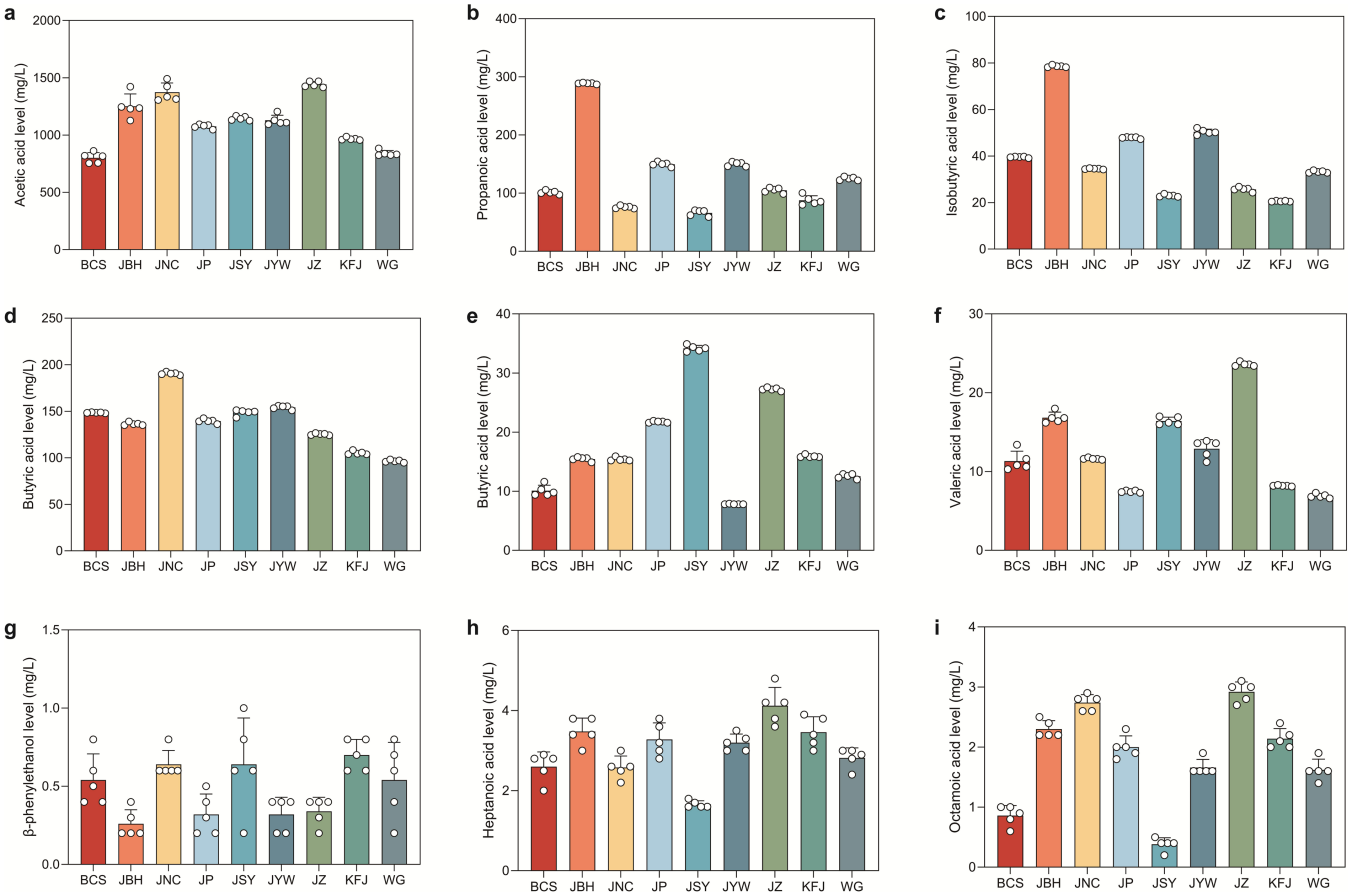
Comparative profiles of organic acids in Huangshui (HS) samples obtained from different regions: **(a)** Acetic acid; **(b)** propionic acid; **(c)** isobutyric acid; **(d)** butyric acid; **(e)** isovaleric acid; **(f)** valeric acid; **(g)** *β*-phenylethanol; **(h)** heptanoic acid; **(i)** octanoic acid.

From a fermentation ecology perspective, these physicochemical differences represent more than simple compositional descriptors; they define the functional boundaries within which microbial guilds assemble and metabolize. Previous studies have suggested that the physicochemical features of HS reflect the functional status of pit fermentation systems and may serve as metabolic fingerprints of the fermentation state (Zhang et al., 2020). In the present study, the coexistence of marked differences in reducing sugars, acidity, ammonium nitrogen, and phosphorus implies a region-dependent variation in substrate turnover, nitrogen cycling, and esterification potential. For example, the combination of elevated acidity, relatively high ester content, and increased ammonium nitrogen concentration in JZ suggests a metabolically active niche associated with intensified aroma-related transformation. By contrast, regions with higher residual carbohydrate content but lower acidity may reflect comparatively different stages or rates of substrate conversion. Therefore, regional HS heterogeneity should be interpreted as ecological niche differentiation within fermentation pits rather than simply as background compositional noise.

The organic acid profiles further reinforced the view that HS is a regionally differentiated metabolic interface. Among the measured acids, lactic acid dominated the nonvolatile acid pool in all samples, reaching 73–93 g/L, far exceeding the concentrations of other acids present. This consistent predominance indicates that lactate-centered acidogenesis is a universal feature of HS, whereas the distribution of other acids considerably varied among the regions. Short-chain acids, such as propionic, isobutyric, and butyric acids, were generally measured at relatively higher levels, whereas medium-chain acids, including heptanoic and octanoic acids, were detected at lower concentrations. This variation suggests that carbon flux is not identically routed across regions. JBH exhibited the highest total organic acid abundance, particularly in lactic, propionic, and isobutyric acids, indicating a strong acidogenic metabolic signature. In contrast, JZ was characterized by comparatively higher levels of valeric, heptanoic, and octanoic acids, which is consistent with enhanced carbon chain-elongation activity through reverse *β*-oxidation-like processes during fermentation (Guo et al., 2024; Kang et al., 2022; Wang et al., 2021; Zhang et al., 2020). In Baijiu ecosystems, this type of metabolic routing generally emerges from the cooperation between acid-producing microorganisms and chain-elongating bacteria, which convert short-chain precursors into medium-chain fatty acids that are closely related to aroma formation (Cheng et al., 2026b; Qiu et al., 2024; Wei et al., 2024). For example, acid-producing bacteria provide precursors, whereas bacteria that reverse the β-oxidation pathway (such as *Clostridium*) are responsible for carbon chain elongation and the synthesis of medium-chain fatty acids (Qiu et al., 2024).

Region-specific metabolic signatures were also evident for selected volatile and precursor compounds. JSY showed a relatively high isovaleric acid level, suggesting differentiation in branched-chain amino acid-related metabolism, whereas BCS favored butyrate-associated metabolism. KFJ contained the highest β-benzyl alcohol content, indicating regional variation in amino acid-derived aromatic transformation. In addition, acetaldehyde, ethyl acetate, glyoxylic acid, and methanol were detected in all samples (Table 1), reflecting the widespread presence of precursor pools relevant to ester synthesis. As esters are the dominant aroma contributors in NXB, differences in acid pools and precursor availability likely correspond to a distinct esterification potential and flavor orientations among the regions (Cheng et al., 2026a; Xu H. L. et al., 2022; Xu Y. Q. et al., 2022). Taken together, these results support the interpretation that regional physicochemical heterogeneity defines distinct HS ecological niches within which microbial communities are exposed to different selective pressures and metabolic constraints. This niche differentiation provides an ecological basis for region-dependent microbiome assembly and metabolite output, as discussed in subsequent sections. In this study, the sample size was small, which may have affected the generalizability of the results; therefore, future research should incorporate larger sample sizes. For example, samples derived from Henan and Jiangsu Provinces in China are necessary as they represent the main production regions of NXB. Furthermore, assessing HS samples obtained from NXB production sites in Sichuan Province (China), including the cities of Yinbin, Luzhou, and Chengdu, which are famous production regions of NXB (Qian et al., 2021; Song et al., 2020), are also necessary, and expanding the sample source will be beneficial to improving the generalizability of the results.

**Table 1:** The main VOCs of different HS samples detected based on GC-FID.

Compounds	CAS	Formula	MW	BCS	KFJ	JSY	WG	JNC	JZ	JP	JYW	JBH
Acetaldehyde	75–07-0	C_2_H_4_O	44.05	11.80 ± 0.87c	4.60 ± 0.53d	12.67 ± 4.05c	13.87 ± 1.40bc	12.00 ± 0.80c	20.00 ± 0.40a	14.13 ± 1.62b	14.20 ± 0.60b	14.87 ± 0.23b
Ethylacetate	141–78-6	C_4_H_8_O_2_	88.10	23.60 ± 0.40e	36.53 ± 0.23b	27.20 ± 0.20d	34.67 ± 0.12bc	31.87 ± 0.31c	24.20 ± 0.00e	36.73 ± 0.12b	45.93 ± 0.50a	35.40 ± 0.20b
Acetal	105–57-7	C_6_H_14_O_2_	118.17	0.00 ± 0.00b	0.00 ± 0.00b	0.00 ± 0.00b	0.00 ± 0.00b	0.00 ± 0.00b	1.47 ± 0.12a	0.33 ± 0.31ab	0.00 ± 0.00b	0.00 ± 0.00b
Methanol	67–56-1	CH_4_O	32.04	48.33 ± 5.87a	42.93 ± 2.84ab	27.87 ± 2.58d	33.40 ± 1.06c	34.60 ± 1.25bc	37.33 ± 1.62bc	34.73 ± 4.11bc	35.40 ± 1.60bc	35.07 ± 0.31bc
Ethylbutyrate	105–54-4	C_6_H_12_O_2_	116.15	0.67 ± 0.12d	1.27 ± 0.12c	5.40 ± 0.00a	0.80 ± 0.00d	1.40 ± 0.00c	1.00 ± 0.00 cd	2.00 ± 0.00b	0.60 ± 0.00d	1.07 ± 0.12 cd
Sec-Butanol	78–92-2	C_4_H_10_O	74.12	0.60 ± 0.00c	0.40 ± 0.00c	1.07 ± 0.12ab	0.00 ± 0.00d	0.93 ± 0.12b	1.20 ± 0.00a	0.60 ± 0.00c	0.00 ± 0.00d	0.60 ± 0.00c
1-Propanol	71–23-8	C_3_H_8_O	60.09	4.60 ± 0.00e	5.20 ± 0.00e	16.20 ± 0.00b	4.53 ± 0.12e	5.33 ± 0.12e	19.33 ± 0.31a	9.40 ± 0.20c	8.93 ± 0.23c	11.07 ± 0.42d
Isobutanol	78–83-1	C_4_H_10_O	74.12	0.00 ± 0.00c	1.73 ± 0.12a	0.47 ± 0.42bc	1.47 ± 0.12ab	0.00 ± 0.00c	1.00 ± 0.00b	1.00 ± 0.00b	1.27 ± 0.12ab	2.00 ± 0.00a
Ethylvalerate	539–82-2	C_7_H_14_O_2_	130.18	0.20 ± 0.35a	0.00 ± 0.00b	1.00 ± 0.00a	0.00 ± 0.00b	0.00 ± 0.00b	0.00 ± 0.00b	0.00 ± 0.00b	0.00 ± 0.00b	0.00 ± 0.00b
1-Butanol	71–36-3	C_4_H_10_O	74.12	4.87 ± 0.12d	4.53 ± 0.12d	27.00 ± 0.20a	6.53 ± 0.12c	7.53 ± 0.12c	9.93 ± 0.12b	10.00 ± 0.00b	5.27 ± 0.12d	5.40 ± 0.00d
Isoamylalcohol	123–51-3	C_5_H_12_O	88.14	1.60 ± 0.00d	4.60 ± 0.00a	2.00 ± 0.00 cd	3.40 ± 0.00b	0.80 ± 0.00e	1.40 ± 0.00b	3.07 ± 0.46d	3.60 ± 0.00bc	3.13 ± 0.12b

Note: The mean ± SD of measurements made in triplicates were used to reflect the characteristic values. Different lowercase letters within the same row indicate significant differences (*p* < 0.05).

### 3.2 Microbial guild assembly reveals acidogenic, methanogenic, and chain-elongating specialization

#### 3.2.1 Community diversity supports the region-dependent ecological structuring of HS microbiota

After quality-filtering, the effective sequence numbers ranged from 57,582–91,907 for bacteria and 79,764–92,671 for fungi (Supplementary Table S1). The rarefaction curves approached saturation with increasing sequencing depth (Supplementary Figure S1), indicating that most of the bacterial and fungal diversity had been captured and that sequencing coverage was adequate for subsequent ecological analyses. HS samples were collected from multiple spatial points within each fermentation pit and homogenized prior to sequencing to improve the representativeness of regional microbial assemblages. Similar sampling strategies had been adopted in previous HS microbiome studies, and amplicon sequencing under standardized workflows exhibited acceptable technical reproducibility (Chen et al., 2021; Jiao et al., 2022).

Alpha diversity analysis indicated clear regional variations in microbial richness and evenness (Supplementary Table S2), supporting the view that HS does not harbor a uniform microbiota across the production regions. For the bacterial communities, JBH and JZ exhibited relatively higher Chao1, ACE, Shannon, and Simpson indices, suggesting a greater richness and more even community organization, whereas JSY displayed comparatively lower bacterial diversity. For the fungal communities, JNC and JP showed higher richness and diversity than the other regions did, indicating more complex fungal assemblages. These results are consistent with previous findings that regional brewing environments, pit age, and fermentation management can significantly influence microbial succession in Baijiu fermentation systems (Guo et al., 2024; Kang et al., 2021).

**Figure 3:**
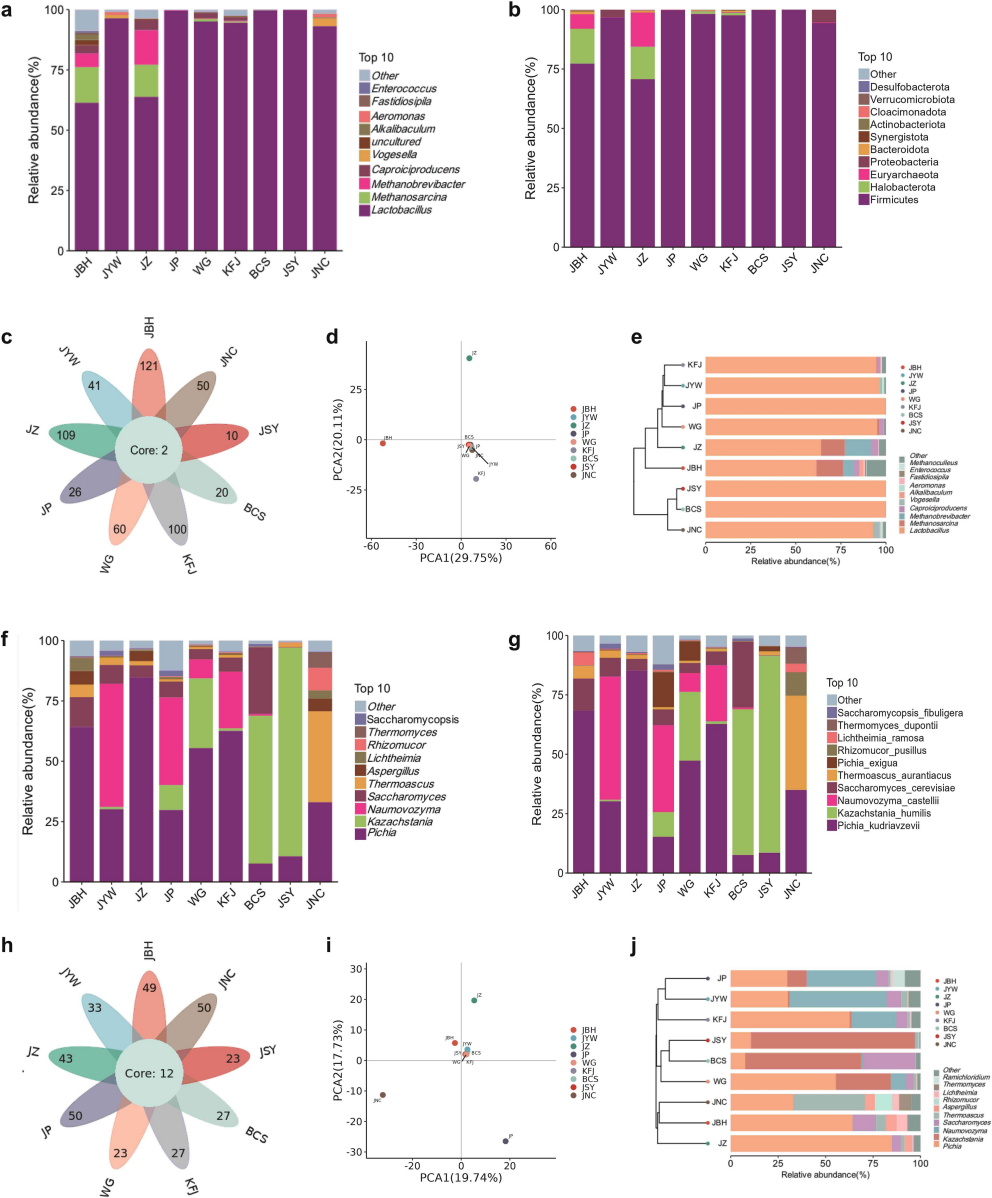
Comparative microbial community structure and diversity patterns in Huangshui (HS) samples. **(a)** Relative abundance of dominant bacterial genera (top 10). **(b)** Relative abundance of dominant bacterial phyla (top 10). **(c)** Venn diagram showing the shared and unique bacterial operational taxonomic units (OTUs) among different samples. **(d)** Principal component analysis (PCA) of the bacterial community structure. **(e)** Hierarchical clustering analysis of the bacterial communities based on genus-level abundance. **(f)** Relative abundance of the dominant fungal genera (top 10). **(g)** Relative abundance of the dominant fungal species (top 10). **(h)** Venn diagram showing the shared and unique fungal OTUs among different samples. **(i)** PCA of the fungal community structure. **(j)** Hierarchical clustering analysis of the fungal communities based on genus-level abundance.

The shared and unique OTU patterns observed further support the strong regional differentiation of HS microbiota. Only two bacterial OTUs were common to all regions (Figure 3c), accounting for 0.37% of the total number of bacterial OTUs identified, whereas JBH contained the highest number of unique bacterial OTUs (121; 22.45%). For fungi, 12 OTUs were shared among all regions (3.56% of the total number of fungal OTUs), whereas JNC and JP had the largest number of region-specific fungal OTUs (50; 14.83%) (Figure 3h). In contrast, JSY exhibited relatively low proportions of both unique bacterial OTUs and fungal-specific taxa. Although OTU-level overlap was limited, the broader ecological message is that HS microbial communities were regionally structured, suggesting that local fermentation environments impose selective pressures that shape community assembly. These region-dependent patterns provide an ecological foundation for functional differentiation among microbial guilds in the context of HS as a transitional niche linking FG and PM. Additionally, the potential intra-regional heterogeneity observed may be substantial given the known variations in pit fermentation microbiota and metabolite profiles.

#### 3.2.2 Bacterial communities indicate specialization toward acidogenesis, methanogenesis, and chain elongation

At the phylum level, the bacterial communities in all HS samples were dominated by Firmicutes, with relative abundances >77%; Halobacterota- and Euryarchaeota-affiliated sequences were also consistently detected. This compositional pattern is consistent with the strongly anaerobic, acidic, and substrate-rich conditions of fermentation pits, where fermentative metabolism and organic-acid accumulation prevail. Rather than indicating simple taxonomic dominance, the bacterial structure of HS suggests an assembly of functionally differentiated guilds adapted to carbon conversion under low-pH conditions. At the genus level, *Lactobacillus*, *Methanosarcina*, *Methanobrevibacter*, and *Caproiciproducens* were the dominant taxa in most regions (Figure 3a). Among these, *Lactobacillus* was overwhelmingly abundant (>61%) in all samples and approached near-complete dominance in JSY. This pattern strongly suggests that acidogenic bacteria form the core bacterial guild in HS. In Baijiu fermentation, *Lactobacillus* is widely recognized for its contribution to lactate accumulation, acid-stress maintenance, and modulation of the acid–ester balance, particularly through indirect effects on ethyl lactate formation (Cheng et al., 2024; Xu Y. Q. et al., 2022). Therefore, its widespread dominance in the present study is consistent with the high lactic acid levels observed across regions and supports the interpretation of HS as a lactate-centered acidic interface (Du et al., 2018).

In addition to acidogenic bacteria, methanogenic archaea-affiliated genera, such as *Methanosarcina* and *Methanobrevibacter*, were repeatedly detected. Their occurrence suggests the presence of a syntrophic guild potentially involved in hydrogen transfer and redox balance under anaerobic fermentation conditions. Although amplicon data alone cannot resolve metabolic directionality, the co-occurrence of methanogenic taxa with fermentative bacteria is consistent with a community structure in which reduced intermediates generated during acidogenesis may be further redistributed through archaeal–bacterial interactions. Such processes have been proposed to stabilize fermentation ecosystems and influence carbon flow in pit-associated microbiomes. C*aproiciproducens*, a representative chain-elongating taxon, was also consistently present, indicating the potential to convert short-chain substrates into medium-chain fatty acids. This observation aligns with the region-specific differences observed for valeric, heptanoic, and octanoic acids (Section 3.1) and suggests that bacterial guild assembly in HS includes not only acid producers but also taxa associated with reverse *β*-oxidation-like metabolism. At the species level, *Lactobacillus acetotolerans* and uncultured *Caloramator*-related sequences were widely distributed across the samples (Figure 3b), further indicating an adaptation to acidic, anaerobic, and thermotolerant pit environments. These taxa can be considered ecological specialists favored by the selective conditions prevailing in HS. Beta diversity analysis revealed a clear regional separation of bacterial communities. The first two PCA axes explained 49.86% of the total variance, with JZ and JBH showing distinct clustering patterns relative to those of the other regions (Figure 3d). Hierarchical clustering at the genus level revealed similarities between JSY and BCS, whereas JNC and JBH exhibited greater divergence (Figure 3e). Taken together, these results suggest that bacterial assembly in HS is not random but rather regionally filtered, giving rise to distinct combinations of acidogenic, methanogenic, and chain-elongating guilds (Guan et al., 2023). As HS occupies the interface between FG and PM, this bacterial restructuring is likely relevant to the redistribution of carbon and generation of flavor-related acid precursors during fermentation (Cheng et al., 2024; Guo et al., 2024).

#### 3.2.3 Fungal communities contribute to substrate depolymerization, fermentation, and precursor supply

Fungal communities displayed a different but equally structured pattern of regional specialization when compared with that of bacteria. At the phylum level, Ascomycota was predominant in all HS samples, with relative abundances >86%, whereas Mucoromycota and Basidiomycota were detected at lower abundances. This predominance is typical of solid-state fermentation ecosystems in which fungi with strong tolerance to acidity, osmotic stress, and fluctuating substrate availability are selectively enriched.

At the genus level, *Pichia*, *Saccharomyces*, *Kazachstania*, *Aspergillus*, and *Naumovozyma* were the major fungal taxa detected in most samples (Figure 3f). From a functional perspective, these genera collectively represent a guild involved in substrate depolymerization, alcoholic fermentation, and aroma precursor generation. Filamentous fungi such as *Aspergillus* can degrade starch and proteins into fermentable sugars, peptides, and amino acids, thereby providing upstream substrates for both bacterial metabolism and flavor compound synthesis (Guo et al., 2024; Jiao et al., 2022; Wang et al., 2021). In contrast, yeasts such as *Saccharomyces*, *Pichia*, and *Kazachstania* are more directly associated with ethanol production, secondary metabolite transformation, and aroma-active precursor formation.

Among the dominant fungal genera, *Pichia* showed particularly pronounced regional variation, reaching high abundance in JZ, whereas its abundance remained much lower in BCS. This pattern suggests that fungal assembly in HS is sensitive to region-specific fermentation environments and may respond differently from that of the bacterial microbiota. Previous studies have reported different dominant fungal genera in HS, including *Cladosporium*, *Aspergillus*, and *Kazachstania*, depending on the sampling site and production conditions (Li et al., 2026; Wei et al., 2024), indicating that fungal communities are highly context dependent. In the present study, the repeated detection of fermentative and hydrolytic fungi supports the idea that HS contains a metabolically relevant fungal guild rather than residual fungal DNA passively washed from FG. At the species level, *Pichia kudriavzevii*, *Saccharomyces cerevisiae*, *Naumovozyma castellii*, and *Kazachstania humilis* were widely distributed across the regions (Figure 3g). These taxa are functionally relevant to Baijiu fermentation because they may contribute to precursor formation, tolerance to stressful late-stage fermentation conditions, and interactions with bacterial partners. For example, *S. cerevisiae* synergistically interacts with acid-producing bacteria, such as *Clostridium tyrobutyricum*, thereby influencing environmental acidity, medium-chain fatty acid production, and ethyl ester synthesis (Guan et al., 2023). Moreover, *P. kudriavzevii* is an aroma-related yeast capable of participating in acid modulation and ester-associated transformation during Baijiu fermentation (Li et al., 2026; Wei et al., 2024). In this context, the fungal community in HS can be interpreted as an upstream functional layer that regulates precursor release and supports downstream bacterial metabolism.

Regional differentiation in fungal community composition was also evident from the beta diversity analysis results. The first two PCA axes explained 37.47% of the total variation, with JZ, JP, and JNC showing a clustering pattern distinct from that of the other regions (Figure 3i). Hierarchical clustering based on genus-level abundance indicated relative similarity between JNC and JBH, whereas JZ and JP showed greater divergence (Figure 3j). Although the fungal communities generally displayed lower dominance complexity than the bacterial communities did, their regional restructuring likely has important metabolic consequences because fungi influence the supply of fermentable carbon, amino acid-derived precursors, and ester-associated intermediates. Therefore, bacterial and fungal communities in HS should not be viewed independently; rather, they form complementary functional guilds in which fungi contribute to substrate depolymerization and precursor generation, whereas bacterial taxa dominate acid production, redox redistribution, and chain elongation. This coordinated guild assembly provides a microbial basis for the region-dependent metabolite routing and flavor differentiation observed in HS (Jiao et al., 2022; Liu et al., 2026; Wang et al., 2021).

### 3.3 Region-specific metabolite routing shapes acid–ester flavor orientation

**Figure 4:**
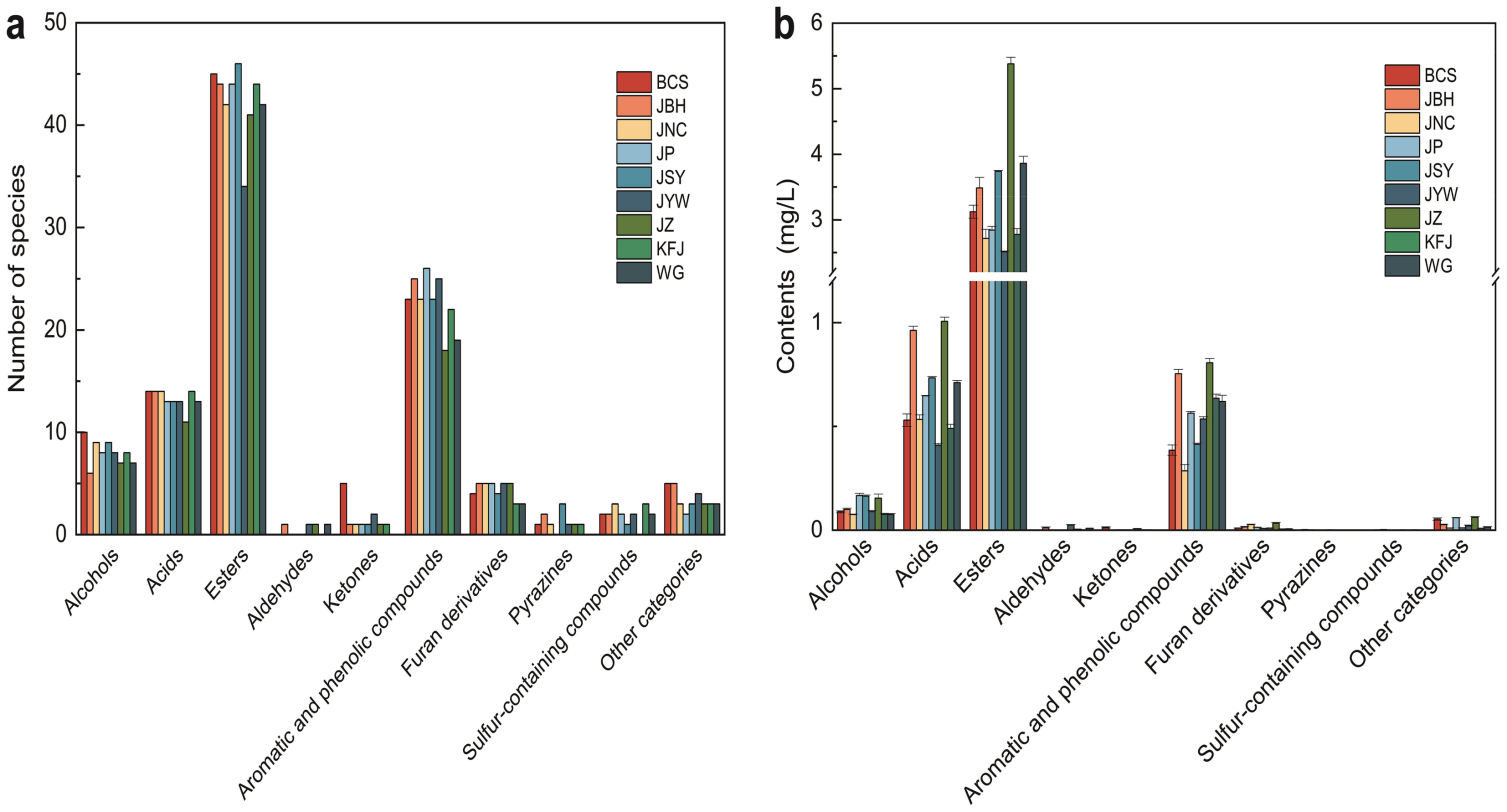
Comparative profiling of volatile flavor compounds in Huangshui (HS) samples. **(a)** Number of identified volatile compounds classified into different chemical categories, including alcohols, acids, esters, aldehydes, ketones, aromatic and phenolic compounds, furan derivatives, pyrazines, sulfur-containing compounds, and other categories. **(b)** Total concentration of volatile compounds in different chemical categories (mg/L).

Volatile metabolite profiling of HS provided further evidence that this matrix functions as an active metabolic interface rather than as a passive repository of fermentation residues. Across all the samples, 162 VOCs were detected using HS-SPME-GC–MS, including esters, alcohols, organic acids, ketones, aldehydes, and aromatic compounds (Figure 4 and Supplementary Table S3). Esters represented the most abundant and diverse class of compounds, followed by organic acids and aromatic derivatives. This overall ester-enriched composition is consistent with the characteristic aroma profile of NXB, in which ester compounds serve as major contributors to sensory quality (Xu Y. Q. et al., 2022). Ethyl hexanoate is particularly recognized as a signature aroma compound of NXB, and its relative balance with ethyl lactate is often considered a practical indicator of flavor quality. From this perspective, the ester-rich nature of HS strongly suggests that it participates in the accumulation of aroma precursors and facilitates a metabolic environment favoring esterification.

Although ester dominance was observed across all the regions, the overall VOC composition showed clear regional differentiation. The PCA results revealed distinct separation trends among the HS samples obtained from different production regions (Supplementary Figure S2), indicating that volatile metabolite structures were not randomly distributed but instead shaped by region-specific fermentation contexts. This differentiation likely reflects the combined effects of physicochemical niche variation, substrate availability, and regionally structured microbial communities. In addition to esters, organic acids constituted another major component group in the volatile fraction, and their coexistence with medium-chain fatty acids further supports the view that HS sustains active carbon conversion processes beyond simple acid accumulation. Rather than representing a static endpoint mixture, the volatile profiles of HS reflect the region-specific routing of carbon toward acidogenesis, chain elongation, and esterification.

Several region-associated metabolite features further support this interpretation. The presence of short-chain acids, together with medium-chain fatty acids, suggests that precursor formation and downstream carbon chain elongation coexist within HS ecosystems, which is consistent with the bacterial guild specialization described in Section 3.2.2. Alcohols were also widely detected, including higher alcohols such as 3-methyl-1-butanol, a compound associated with fruity notes and previously proposed as a geographic discrimination marker in Baijiu (Song et al., 2020). Its detection in HS implies that this interface may retain region-specific aromatic signatures linked to amino-acid catabolism and increased alcohol metabolism. Similarly, the presence of compounds such as acetaldehyde, ethyl acetate, glyoxylic acid, and methanol indicates that HS contains metabolically relevant precursor pools connected to ester synthesis and aroma diversification. These precursor- and product-level differences suggest that HS derived from different production regions does not share a uniform flavor-forming capacity but instead exhibits differentiated metabolic potential.

The VOC data support a flavor-oriented interpretation of HS metabolism. In some regions, the metabolite output appeared biased toward acid accumulation, whereas in others, the more pronounced formation of medium-chain acids and ester-associated compounds suggested stronger carbon transfer toward aroma-relevant pathways. Such differences are highly relevant to NXB production because flavor quality depends not only on the abundance of individual compounds but also on the coordinated balance between acid pools, alcohol precursors, and esterification products. Therefore, regional variations in the HS VOC composition can be understood as a chemical manifestation of differentiated metabolite routing within distinct fermentation niches. This variation in composition also explains why HS obtained from different production regions may differ in practical applications, such as esterification solution preparation, PM maintenance, and seasoning Baijiu production (Gao et al., 2023). In this sense, HS serves not only as a by-product containing volatile compounds but also as a chemically dynamic interface that records and potentially influences region-specific flavor orientation during NXB fermentation. Previous studies have identified many key flavor compounds and factors influencing Baijiu quality, including the application of isolated functional strains during fermentation (Guan et al., 2023; Zou et al., 2018). However, factors influencing the flavor of most types of Baijiu, such as the effects of HS on the flavor links of NXB, FG, and PM during fermentation, remain unclear (Guo et al., 2024; Kang et al., 2022). Future studies should investigate the effects that HS has on the flavor links of NXB, FG, and PM, increase the sample size of HS obtained from different origins, and clarify the flavor-metabolism characteristics of HS collected during different fermentation times.

### 3.4 Physicochemical filtering reorganizes microbiome–metabolome coupling at the HS interface

**Figure 5:**
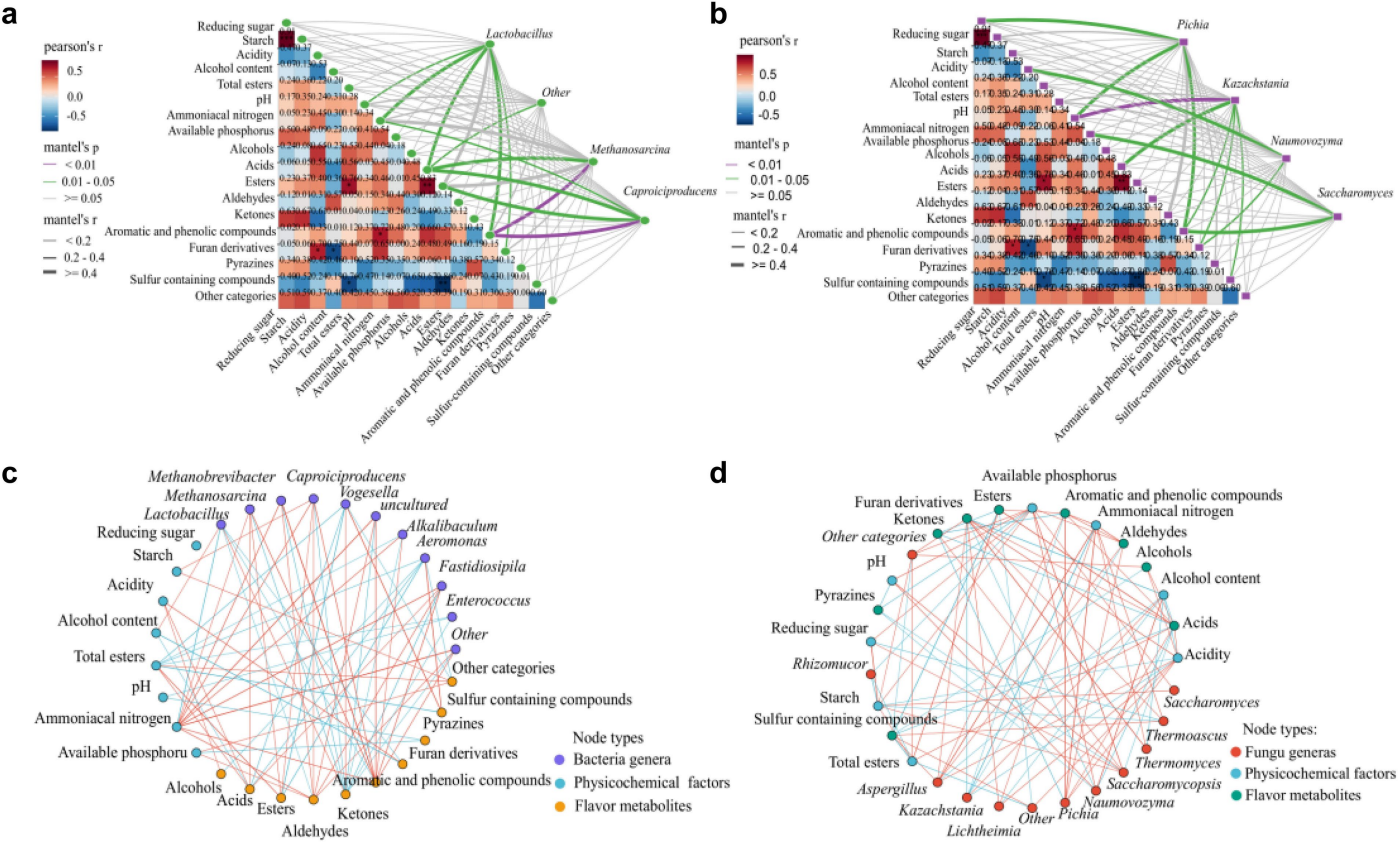
Correlation analysis and interaction networks among the microbial communities, physicochemical properties, and flavor metabolites in Huangshui (HS). **(a)** Pearson correlation heatmap showing relationships among the physicochemical parameters and flavor metabolites, combined with Mantel test-based association analysis between the bacterial genera and environmental factors. **(b)** Pearson correlation heatmap combined with Mantel test analysis showing associations between the fungal genera and physicochemical/flavor variables. **(c)** Network analysis illustrating correlations among the bacterial genera, physicochemical factors, and flavor metabolites (red lines indicate positive correlations; blue lines indicate negative correlations). **(d)** Network analysis illustrating correlations among the fungal genera, physicochemical factors, and flavor metabolites. Note: Edge color represents correlation direction, edge thickness indicates correlation strength, and *p* < 0.05 indicates the statistical significance.

As HS accumulates at the bottom of the fermentation pits and remains in contact with both PM and FG, it may function as an ecological and metabolic interface linking pit microecology with flavor metabolism in FG (Cheng et al., 2025a; Zhang X. Y. et al., 2024; Hu et al., 2021). Presently, most studies on HS have been largely descriptive, with limited emphasis on the integrated ecological relationships of HS in fermentation systems (Gao et al., 2023; Guo et al., 2024). Additionally, how production regions reshape the coupled physicochemical, microbial, and metabolic features of HS in the NXB fermentation system remains unclear; in particular, the ecological associations and metabolic networks linking HS microbiota to flavor metabolite differentiation across different regions have yet to be characterized (Cheng et al., 2025a; Guo et al., 2024). In the present study, Mantel tests and co-occurrence network analyses were performed to further examine how physicochemical heterogeneity is linked to microbial assembly and volatile metabolite differentiation (Figure 5). Overall, the integrated results revealed nonrandom associations among environmental variables, dominant microbial taxa, and aroma-related metabolites, supporting the view that HS functions as a coordinated ecological interface rather than as a passive fermentation by-product. In particular, acidity, nitrogen availability, and organic acid composition were closely associated with both microbial community structure and volatile metabolite patterns, suggesting that regional physicochemical conditions act as ecological filters that constrain microbiome–metabolome coupling within HS.

#### 3.4.1 Bacterial genus associations support acidogenesis–esterification–chain elongation coupling

Within the bacterial association network (Figures 5a,c), *Lactobacillus* emerged as one of the most connected nodes and showed multiple associations with ester compounds. Given the established role of *Lactobacillus* in lactic acid production and acid–ester balance regulation during Baijiu fermentation (Xu Y. Q. et al., 2022), its central position in the present network was consistent with a core ecological role in HS. Rather than acting solely as an acid producer, *Lactobacillus* may contribute to shaping the metabolic context in which esterification occurs, particularly through its influence on lactate accumulation and the availability of substrates related to ethyl lactate and ethyl acetate formation. Previous simulated fermentation studies have shown that reinforcement with specific *Lactobacillus* strains can shift the ratios of these key esters, thereby altering the major aroma indicators of Baijiu (Cheng et al., 2025b; Xu Y. Q. et al., 2022). Although the present data are correlational, the observed network structure supports the interpretation that lactic acid bacteria are closely linked to downstream aroma-related metabolic balance in HS.

In addition to *Lactobacillus*, methanogenic genera and chain-elongating bacteria, including *Caproiciproducens*, were embedded in the bacterial association network. Their occurrence in relation to acid metabolites is consistent with the ecological model proposed above, in which acidogenesis, hydrogen transfer, and medium-chain fatty acid formation are functionally connected within HS. *Caproiciproducens* is widely associated with chain elongation and the production of medium-chain carboxylic acids, and its placement within the network is, therefore, compatible with the regional differentiation observed for valeric, heptanoic, and octanoic acids (Section 3.1). Similarly, archaeal-affiliated methanogenic taxa may reflect syntrophic interactions involved in redox redistribution under anaerobic fermentation conditions. Although these associations do not demonstrate direct metabolite exchange, they support the view that HS harbors bacterial consortia capable of coordinating acid production, reducing intermediate turnover, and facilitating carbon chain extension.

Other bacterial genera, including *Aeromonas* and *Fastidiosipila*, were also associated with ester, sulfur-containing compound, and ketone production. These patterns suggest that the bacterial network in HS is not limited to dominant acidogenic taxa but also includes subordinate genera that may participate in specialized branches of aroma metabolism or substrate competition. A previous study reported a negative relationship between *Fastidiosipila* and ester abundance (Zhou et al., 2024), which is broadly consistent with the present results. Taken together, the bacterial network supports a model in which HS-associated bacterial guilds are metabolically differentiated yet interconnected, thus collectively contributing to acid accumulation, precursor redistribution, and aroma-oriented metabolite output.

#### 3.4.2 Fungal genus associations indicate specialization in precursor transformation and fermentation-stage adaptation

Compared with the bacterial network, the fungal association network (Figures 5b,d) displayed lower overall connectivity but still revealed functionally meaningful patterns among the dominant yeast genera. *Saccharomyces* was primarily associated with acid-related metabolites, consistent with its role in alcoholic fermentation and precursor transformation in solid-state fermentation systems. Rather than directly explaining the specific aroma products produced, this pattern suggests that *Saccharomyces* may influence the upstream metabolic environment by modulating ethanol formation, carbon availability, and the biochemical context in which acid and ester metabolism proceeds.

*Kazachstania* was more selective and, in some cases, showed negative correlations with certain metabolites, implying an ecological niche differentiation within the yeast community. Such differentiation may reflect competition for substrates or differing tolerance to acid and ethanol stress, both of which likely influence fungal persistence in HS. *Naumovozyma* was positively associated with alcohol content. This relationship should be interpreted cautiously; rather than indicating maximal ethanol-producing capacity, the ecological persistence of *Naumovozyma* under relatively high-ethanol fermentation conditions may be reflected. Moreover, *N. castellii* retains metabolic activity during the late fermentation stages when elevated ethanol concentrations suppress the growth of other microorganisms (Zhang Y. L. et al., 2024). Therefore, its abundance in HS may serve as an indicator of late-stage fermentation ecology rather than as a direct driver of ethanol accumulation.

*Pichia* was also associated with both acid- and ester-related metabolites, supporting its potential relevance to aroma modulation in HS. This interpretation is consistent with previous reports that *P. kudriavzevii* can promote the formation of compounds such as phenethyl acetate in fermentation systems (Chen Y. et al., 2025; Tsaruk et al., 2025). Therefore, in the present study, the fungal network supports the view that yeasts are functionally specialized members of the HS microbiota, contributing not only to fermentation but also to precursor transformation and aroma-related metabolic diversification. Although the fungal associations were less densely connected than the bacterial ones were, they indicated that fungal assemblages may indirectly influence flavor formation by regulating substrate release, alcohol metabolism, and intermediate precursor pools.

#### 3.4.3 Integrated ecological interpretation of microbiome–metabolome coupling in HS

Taken together, the Mantel and network analyses results suggest that region-dependent physicochemical variations restructure both microbial guild composition and metabolite output at the HS interface. Among the measured environmental variables, acidity, nitrogen availability, and organic acid pools showed particularly strong associations with microbial composition and volatile profiles, indicating that they likely function as key ecological filters. Additionally, *Lactobacillus* emerged as a central taxon associated with acid–ester balance, whereas methanogenic and chain-elongating taxa were linked to carbon redistribution and medium-chain fatty acid formation. Under these selective conditions, bacterial taxa were predominantly involved in acid production, redox-linked carbon redistribution, and chain elongation, whereas fungal communities primarily contributed to substrate depolymerization, fermentation-stage adaptation, and precursor transformation. The coordinated interactions between these guilds provide a plausible ecological explanation for the region-specific VOC patterns described in Section 3.3.

Importantly, the regional differentiation observed in HS likely did not arise from the isolated dominance of a single taxon but rather from the reorganization of microbiome–metabolome coupling under different physicochemical regimes. In other words, the regional flavor orientation in HS is interpreted more reasonably as an emergent property of filtered community assembly and coordinated metabolic routing rather than as the outcome of one dominant microorganism. This perspective reinforces the concept of HS as a dynamic metabolic hub linking FG, PM, and aroma-related transformations during NXB fermentation.

Notably, the limitations of the present analysis should be acknowledged. This was an observational and cross-sectional study based on endpoint HS samples collected from multiple production regions, and no perturbation experiments were performed. Therefore, the ecological networks described herein are correlation-based and cannot demonstrate the causality or directionality of metabolite flux. Nevertheless, by integrating physicochemical characterization, microbiome profiling, and volatile metabolomics across multiple regions, this study establishes a systematic baseline for understanding HS-mediated ecological differentiation during Baijiu fermentation. The inferred links among FG, HS, and PM should be regarded as testable hypotheses that warrant further validation through time-series sampling, metatranscriptomics, metabolite flux analysis, and controlled microcosm experiments.

### 3.5 Implications for fermentation monitoring, origin discrimination, and precision regulation

Previous studies have shown that microbial community assembly and flavor metabolism during Baijiu fermentation are jointly regulated by environmental conditions, pit characteristics, fermentation periods, and functional microorganisms (Cheng et al., 2026b; Tong et al., 2025). This joint regulation is true in HS as well, where certain differences in production regions and processes also influence microbial community assembly and flavor metabolism. Beyond its ecological significance, the present study highlights the practical relevance of HS for understanding and managing NXB fermentation. As HS integrates physicochemical conditions, microbial guild composition, and aroma-associated metabolites at the interface between FG and PM, it can be regarded as an informative window into fermentation status rather than merely as a residual liquid fraction. Therefore, variables such as acidity, ammonium nitrogen, available phosphorus, lactate abundance, and ester-related metabolite patterns may be useful composite indicators for evaluating pit activity and monitoring fermentation performance.

The marked regional differentiation observed in HS further suggests that this interface retains origin-related ecological signatures. Distinct combinations of physicochemical properties, microbial communities, and volatile metabolites were consistently associated with different production regions, indicating that HS may provide a complementary basis for origin discrimination alongside conventional criteria, such as geographical indications, production records, and sensory characteristics. Although no predictive classification model was established in the present study, the region-specific microbiome–metabolome patterns identified herein support the feasibility of using HS-associated fingerprints for regional characterization.

More importantly, the ecological filtering framework proposed in this study offers a basis for precise fermentation during NXB production. The observed links among acidity, nutrient availability, functional microbial guilds, and acid–ester balance suggest that HS may represent a tractable node for regulating flavor-oriented metabolism. The manipulation of environmental conditions or selective enrichment of acidogenic, chain-elongating, or aroma-associated taxa could potentially influence carbon routing and precursor formation during pit fermentation. Nevertheless, these implications should be interpreted with caution because the present study was based on cross-sectional and correlational evidence rather than on controlled intervention experiments. Further validation through time-series monitoring, targeted microbial reinforcement, and functional verification is required before HS-based precision regulation can be translated into practical process control.

## 4 Conclusion

In the NXB fermentation system, HS should be considered an active ecological and metabolic interface rather than as a simple liquid by-product. Across the nine production regions included, distinct physicochemical niches in HS were associated with the region-dependent assembly of acidogenic bacteria, methanogenic archaea, chain-elongating taxa, and fermentative yeasts, which together reshaped the volatile metabolite output and acid–ester balance. These findings support a model in which regional physicochemical filtering reorganizes microbiome–metabolome coupling at the HS interface, thereby contributing to flavor differentiation. From an applied perspective, HS-associated physicochemical and microbiome–metabolome fingerprints may provide useful indicators for fermentation-state monitoring, origin discrimination, and the precise regulation of aroma formation in NXB production.

The quality of NXB is determined by the coordinated ecological and metabolic interactions occurring during fermentation, including among the HS, FG, and PM systems, as well as interactions involving physicochemical properties, the microbiome, and metabolome. HS is regarded as a bridge linking the microbiome and metabolome in FG and PM, and it plays a vital role in fermentation systems. Future studies integrating time-series sampling, high-resolution sequencing, and functional validation are necessary to verify the causal pathways inferred in the present study. Understanding how HS links the coupled physicochemical, microbial, and metabolic features of PM and FG is beneficial for understanding the role of HS in fermentation systems and could facilitate the development of precision fermentation strategies for NXB, such as those involving the isolation and application of functional microorganisms in HS.

## Data Availability

The datasets generated and analyzed during the current study are also available from the corresponding author upon request. The data presented in this study were deposited in the National Center for Biotechnology Information (NCBI) repository (Accession number: PRJNA1397324 and PRJNA1397518).
