# Supplementary material for: Regional physicochemical filtering shapes microbiome–metabolome coupling at the Huangshui interface during Nongxiangxing Baijiu fermentation
Source: Front Microbiol. 2026 Jun 10;17:1869375. doi: 10.3389/fmicb.2026.1869375 (PMC13290848; doi:10.3389/fmicb.2026.1869375)
Supplement: Supplementary file 1 [file Table_1.DOCX]

Supplementary Material

# Supplementary Figures and Tables

## Supplementary Figures

**
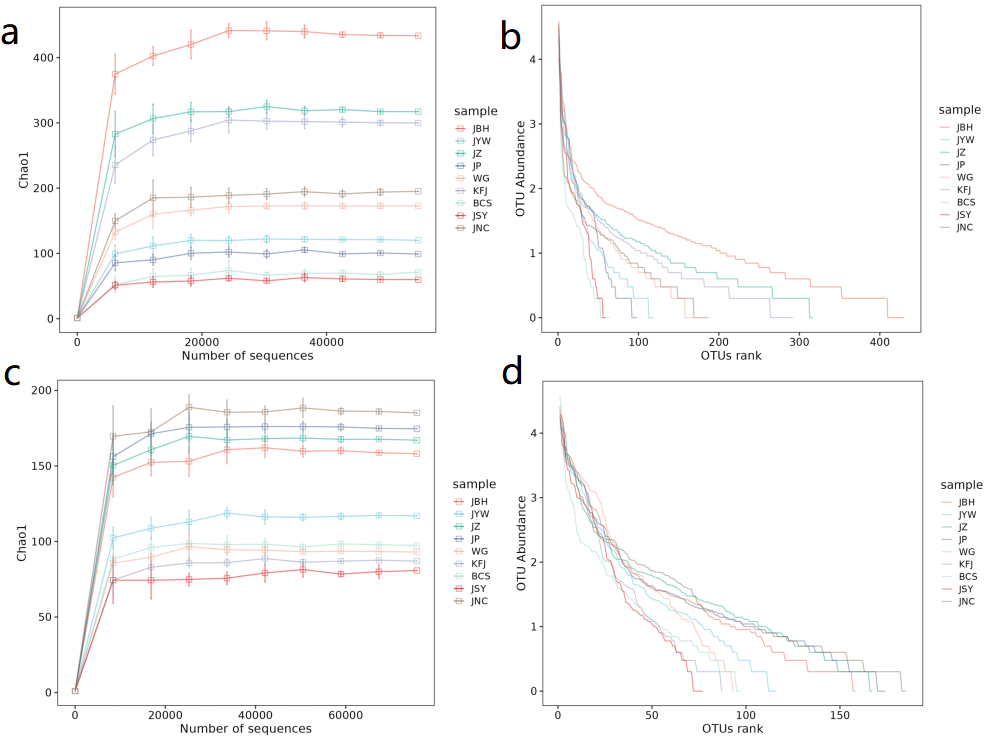
**

**Supplementary Figure S1.** The rarefaction curves and abundance grade curves of sequencing. (a). the rarefaction curves of bacteria sequencing; (b). the abundance grade curve of bacteria sequencing; (c). the rarefaction curves of fungi sequencing; (d). the abundance grade curve of fungi sequencing.

**
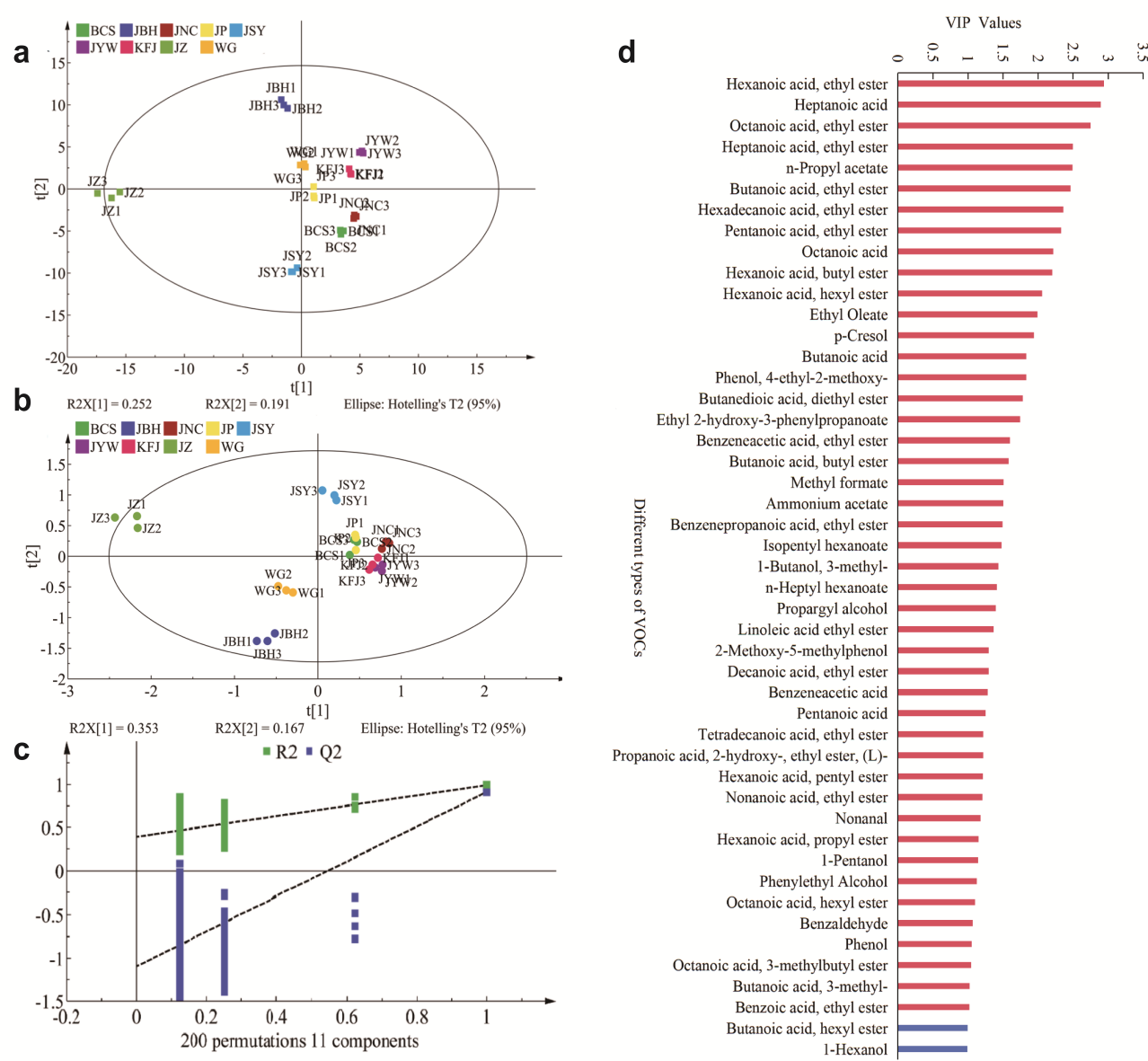
**

**Supplementary Figure S2.** The PCA (A), PLS-DA (B), Hotelling's T2 (C), and (D) VIP analyses based on VOCs detected via HS-SPME-GC-MS.

## Supplementary Tables

**Supplementary Table S1.** The ASV and effective Seqs number of bacteria and fungi in HS samples.

| HS Samples | Bacteria | | Fungal | |
| --- | --- | --- | --- | --- |
|  | ASV_num | Seqs_Num | ASV_num | Seqs_Num |
| JBH | 431 | 70727 | 159 | 87083 |
| JYW | 120 | 76395 | 116 | 88229 |
| JZ | 317 | 57582 | 167 | 87228 |
| JP | 99 | 83590 | 175 | 83851 |
| WG | 171 | 68836 | 93 | 87469 |
| KFJ | 303 | 84158 | 88 | 88063 |
| BCS | 66 | 89493 | 100 | 90960 |
| JSY | 59 | 83623 | 77 | 92671 |
| JNC | 193 | 91907 | 185 | 79764 |
| Total | 1759 | 706311 | 1160 | 785318 |

**Supplementary Table S2.**  The alpha diversity indices of the microbial community in HS samples.

| HS Samples | Bacteria | | | | Fungal | | | |
| --- | --- | --- | --- | --- | --- | --- | --- | --- |
|  | Chao 1 | ACE | Shannon | Simpson | Chao 1 | ACE | Shannon | Simpson |
| JBH | 433.62 | 438.40 | 4.81 | 0.91 | 158.04 | 158.86 | 4.31 | 0.92 |
| JYW | 120.05 | 121.81 | 3.05 | 0.80 | 117 | 118.25 | 3.88 | 0.88 |
| JZ | 317.21 | 318.45 | 3.65 | 0.82 | 167.06 | 167.69 | 4.03 | 0.89 |
| JP | 99 | 101.90 | 2.73 | 0.66 | 174.62 | 175.64 | 4.18 | 0.89 |
| WG | 172.66 | 173.65 | 3.47 | 0.83 | 93 | 93.28 | 4.37 | 0.92 |
| KFJ | 299.80 | 307.11 | 2.85 | 0.71 | 87 | 87.38 | 3.79 | 0.88 |
| BCS | 71.33 | 75.29 | 1.25 | 0.41 | 97.30 | 97.96 | 2.33 | 0.67 |
| JSY | 59.75 | 61.63 | 1.86 | 0.52 | 80.75 | 80.12 | 3.35 | 0.84 |
| JNC | 195.14 | 196.37 | 2.02 | 0.59 | 185.14 | 185.90 | 3.96 | 0.85 |

**Supplementary Table S3.** Relative contents (mg/L) of VOCs in different HS samples.

| **Compounds** | **CAS N.** | **RI-Lib** | **RI** | **BCS** | **JBH** | **JNC** | **JP** | **JSY** | **JYW** | **JZ** | **KFJ** | **WG** |
| --- | --- | --- | --- | --- | --- | --- | --- | --- | --- | --- | --- | --- |
| **Alcohols** |  |  |  |  |  |  |  |  |  |  |  |  |
| 1-Butanol | 71-36-3 | 659 | 1168 | 0.0078±0.0001 | 0.0081±0.001 | 0.0127±0.0003 | 0.0177±0.0001 | 0.0491±0.0032 | 0.0079±0.0002 | 0.0181±0.0013 | 0.0065±0.0002 | 0.0107±0.0001 |
| 1-Pentanol | 71-41-0 | 765 | 1263 | 0.0143±0.0014 | n.d. | 0.0036±0.0004 | 0.0156±0.0115 | 0.0143±0.0006 | 0.0297±0.0016 | 0.0061±0.0005 | n.d. | n.d. |
| 2-Heptanol | 543-49-7 | 900 | 1331 | 0.0004±0 | n.d. | 0.001±0 | n.d. | 0.0006±0 | 0.0006±0 | n.d. | 0.0009±0 | 0.0008±0 |
| 1-Hexanol | 111-27-3 | 868 | 1365 | 0.0439±0.001 | 0.0351±0.0022 | 0.0446±0.0002 | 0.0683±0.0025 | 0.0784±0.0031 | 0.0421±0.0004 | 0.0837±0.0051 | 0.0376±0.0001 | 0.044±0.0003 |
| 1-Heptanol | 111-70-6 | 970 | 1467 | 0.0075±0.0001 | 0.0039±0.0001 | 0.002±0.0001 | 0.0046±0.0001 | 0.0058±0.0003 | 0.0025±0.0001 | 0.0093±0.0007 | 0.0039±0 | 0.0028±0.0001 |
| 2-Nonanol | 628-99-9 | 1102 | 1529 | 0.0005±0 | n.d. | n.d. | 0.0007±0 | n.d. | 0.0008±0 | n.d. | 0.0007±0 | n.d. |
| 2,3-Butanediol, [R-(R*,R*)]- | 24347-58-8 | 743 | 1559 | 0.0029±0.0001 | 0.0051±0.0001 | 0.0014±0.0001 | 0.0021±0.0003 | 0.001±0.0001 | 0.0023±0 | n.d. | n.d. | 0.0039±0.0003 |
| 1-Octanol | 111-87-5 | 1070 | 1568 | 0.0071±0.0003 | 0.0213±0.0006 | 0.0019±0 | 0.0131±0.0002 | 0.0084±0.0004 | 0.0036±0.0001 | 0.0209±0.0013 | 0.0096±0.0001 | 0.0097±0.0001 |
| 2,3-Butanediol | 513-85-9 | 788 | 1596 | 0.0009±0.0001 | n.d. | 0.0017±0.0003 | n.d. | 0.0008±0 | n.d. | 0.0011±0.0003 | 0.0004±0 | 0.0038±0.0004 |
| .alpha.-Terpineol | 98-55-5 | 1189 | 1706 | 0.0027±0 | n.d. | n.d. | n.d. | 0.0052±0.0002 | n.d. | n.d. | n.d. | n.d. |
| 1-Butanol, 3-methyl- | 123-51-3 | 736 | 1223 | n.d. | 0.0264±0.0011 | 0.0065±0.0003 | n.d. | n.d. | n.d. | 0.0147±0.0018 | 0.0173±0.0198 | n.d. |
| Propargyl alcohol | 107-19-7 | 560 | 1875 | n.d. | n.d. | n.d. | 0.0446±0.0016 | n.d. | n.d. | n.d. | n.d. | n.d. |
| **Acids** |  |  |  |  |  |  |  |  |  |  |  |  |
| Heptanoic acid | 111-14-8 | 1080 | 1243 | 0.1469±0.0025 | 0.1653±0.0077 | 0.1066±0.0011 | 0.101±0.0046 | 0.136±0.0063 | 0.0843±0.0029 | 0.2132±0.0088 | 0.078±0.0005 | 0.1163±0.0011 |
| Propanoic acid | 29102 | 706 | 1565 | 0.0016±0.0002 | 0.0052±0.0005 | 0.0042±0.0002 | 0.0024±0.0003 | 0.0071±0.0001 | 0.0079±0.0001 | 0.0154±0.0032 | 0.0063±0.0001 | n.d. |
| Butanoic acid | 107-92-6 | 802 | 1647 | 0.0985±0.0034 | 0.1453±0.007 | 0.1428±0.0041 | 0.1885±0.0048 | 0.2657±0.0221 | 0.0934±0.0016 | 0.2242±0.0119 | 0.139±0.0016 | 0.1266±0.0009 |
| Butanoic acid, 3-methyl- | 503-74-2 | 850 | 1693 | 0.0122±0.0003 | n.d. | n.d. | n.d. | n.d. | n.d. | n.d. | 0.0189±0.0011 | 0.0147±0.0025 |
| Butanoic acid, 2-methyl- | 116-53-0 | 861 | 1693 | 0.0125±0 | n.d. | 0.015±0.0004 | n.d. | n.d. | n.d. | n.d. | n.d. | n.d. |
| Pentanoic acid | 109-52-4 | 901 | 1766 | 0.0956±0.0045 | 0.0841±0.0045 | 0.0988±0.0022 | 0.0651±0.0023 | 0.1284±0.0083 | 0.061±0.0009 | 0.1627±0.0087 | 0.0729±0.0002 | 0.0703±0.0008 |
| Pentanoic acid, 4-methyl- | 646-07-1 | 949 | 1830 | 0.0013±0 | 0.007±0.0003 | 0.0031±0.0001 | 0.0035±0.0002 | 0.0024±0.0003 | 0.0015±0.0001 | n.d. | 0.0025±0.0001 | 0.0039±0.0001 |
| Octanoic acid | 124-07-2 | 1180 | 2087 | 0.1397±0.002 | 0.4249±0.0252 | 0.1279±0.001 | 0.2276±0.0088 | 0.1702±0.0044 | 0.1016±0.001 | 0.2675±0.021 | 0.1414±0.0054 | 0.2859±0.0064 |
| Nonanoic acid | 112-05-0 | 1273 | 2183 | 0.0028±0.0004 | 0.036±0.001 | 0.0027±0.0001 | 0.0109±0.0147 | 0.0017±0 | 0.0097±0.0009 | 0.0167±0.005 | 0.0034±0.0006 | 0.0214±0.0013 |
| n-Decanoic acid | 334-48-5 | 1372 | 2272 | 0.0023±0.0004 | 0.0221±0.0005 | 0.0017±0.0001 | 0.0069±0 | 0.0044±0.0001 | 0.0078±0.0002 | n.d. | 0.0048±0.001 | 0.0257±0.0023 |
| Benzoic acid | 65-85-0 | 1177 | 2408 | 0.0067±0.0001 | 0.0205±0.0014 | 0.0177±0.0004 | 0.0089±0 | 0.0043±0.0001 | 0.0248±0.0026 | 0.0277±0.0052 | 0.0103±0.001 | 0.0171±0.0007 |
| Dodecanoic acid | 143-07-7 | 1567 | 2432 | 0.0017±0.0004 | 0.0052±0.0003 | 0.0017±0 | 0.0028±0.0003 | 0.0017±0.0002 | 0.0045±0.0001 | 0.0048±0.0005 | 0.0022±0.0004 | 0.0038±0.0001 |
| Hydrocinnamic acid | 501-52-0 | 1356 | 2562 | 0.0028±0.0003 | 0.0276±0.0019 | 0.0067±0.0002 | 0.0036±0.0001 | 0.0044±0.0002 | 0.0037±0.0004 | 0.0292±0.0064 | 0.0046±0.0002 | 0.0078±0.0002 |
| Tetradecanoic acid | 544-63-8 | 1768 | 2617 | 0.0051±0.0009 | 0.0113±0.0017 | 0.0026±0.0002 | 0.0224±0.0011 | 0.0032±0.0003 | 0.0076±0.0003 | 0.0109±0.0029 | 0.0035±0.0004 | 0.0156±0.0018 |
| Propanoic acid, 2-methyl- | 79-31-2 | 765 | 1593 | n.d. | 0.0052±0 | n.d. | 0.0033±0.0003 | 0.0041±0.0004 | n.d. | 0.0335±0.041 | n.d. | n.d. |
| 5-Methylhexanoic acid | 628-46-6 | 1057 | 1932 | n.d. | 0.0024±0.0001 | 0.0015±0 | n.d. | n.d. | 0.0013±0 | n.d. | 0.0017±0.0002 | 0.0021±0.0001 |
| **Esters** |  |  |  |  |  |  |  |  |  |  |  |  |
| Butanoic acid, ethyl ester | 105-54-4 | 802 | 1046 | 0.1173±0.0014 | 0.1612±0.0059 | 0.2231±0.0094 | 0.2146±0.0141 | 0.4365±0.015 | 0.1143±0.0033 | 0.1346±0.0026 | 0.1731±0.0043 | 0.1387±0.0018 |
| Butanoic acid, 2-methyl-, ethyl ester | 7452-79-1 | 849 | 1058 | 0.0008±0 | 0.0024±0.0003 | 0.0017±0.0002 | 0.0009±0 | 0.0017±0.0002 | 0.0011±0.0001 | n.d. | 0.0012±0 | 0.001±0.0001 |
| Butanoic acid, 3-methyl-, ethyl ester | 108-64-5 | 853 | 1072 | 0.0012±0.0001 | 0.0038±0.0001 | 0.0055±0.0001 | 0.0013±0.0002 | 0.0021±0.0003 | 0.0021±0.0001 | n.d. | 0.0026±0.0001 | 0.0016±0 |
| Acetic acid, butyl ester | 123-86-4 | 812 | 1076 | 0.0012±0 | n.d. | 0.0037±0.0003 | 0.0039±0 | 0.0105±0.001 | n.d. | 0.0032±0.0002 | n.d. | n.d. |
| Butanoic acid, 3-methylbutyl ester | 106-27-4 | 1056 | 1118 | 0.0025±0 | 0.0046±0.0001 | n.d. | 0.003±0.0002 | n.d. | n.d. | 0.0038±0.0002 | 0.0047±0.0001 | 0.0056±0.0003 |
| Pentanoic acid, ethyl ester | 539-82-2 | 900 | 1130 | 0.272±0.011 | 0.1557±0.0086 | 0.1966±0.0325 | 0.1187±0.0008 | 0.1307±0.0208 | 0.1487±0.0032 | 0.198±0.0107 | 0.1408±0.0053 | 0.0597±0.0039 |
| Pentanoic acid, 4-methyl-, ethyl ester | 25415-67-2 | 969 | 1185 | 0.01±0.0019 | 0.009±0.0008 | 0.0081±0.0007 | 0.0062±0.0003 | 0.0103±0.0015 | n.d. | 0.0257±0.0013 | 0.0041±0.0004 | 0.0047±0.0001 |
| Hexanoic acid, ethyl ester | 123-66-0 | 999 | 1246 | 1.2214±0.2408 | 1.2936±0.1537 | 1.2077±0.1231 | 1.1002±0.0384 | 1.212±0.1216 | 1.2615±0.0653 | 1.3131±0.0171 | 1.218±0.1529 | 1.4731±0.1146 |
| Acetic acid, hexyl ester | 142-92-7 | 1011 | 1273 | 0.0068±0.0003 | 0.0114±0.0008 | 0.0125±0.0004 | 0.014±0.0011 | 0.0162±0.0011 | 0.0126±0.0005 | 0.0268±0.0016 | 0.0074±0.0002 | 0.0102±0.0001 |
| Ethyl 5-methylhexanoate | 10236-10-9 | 1068 | 1287 | 0.0025±0.0002 | 0.01±0.0003 | 0.0028±0.0002 | 0.0017±0.0001 | n.d. | 0.0034±0 | 0.0023±0.0003 | 0.0027±0 | 0.0036±0 |
| Pentanoic acid, butyl ester | 591-68-4 | 1093 | 1315 | 0.0023±0.0002 | 0.0025±0 | 0.0045±0.0001 | 0.0024±0.0002 | 0.0339±0.0002 | 0.0025±0.0002 | 0.0127±0.001 | 0.0012±0.0001 | 0.0045±0.0002 |
| Propanoic acid, 2-methyl-, pentyl ester | 2445-72-9 | 1057 | 1316 | 0.0016±0.0001 | 0.0008±0 | 0.0008±0 | 0.0007±0 | 0.0117±0.0012 | n.d. | n.d. | 0.0002±0 | 0.0008±0 |
| Hexanoic acid, propyl ester | 626-77-7 | 1094 | 1319 | 0.0184±0.0013 | 0.0433±0.004 | 0.0335±0.0013 | 0.0196±0.0016 | 0.0779±0.005 | 0.0318±0.0014 | 0.1389±0.011 | 0.0176±0.0008 | 0.0376±0.0012 |
| Heptanoic acid, ethyl ester | 106-30-9 | 1098 | 1337 | 0.3401±0.0162 | 0.3758±0.0263 | 0.1466±0.0044 | 0.1519±0.0095 | 0.2506±0.0174 | 0.1971±0.0126 | 0.4843±0.0203 | 0.1708±0.0116 | 0.2652±0.0079 |
| Propanoic acid, hexyl ester | 2445-76-3 | 1108 | 1340 | 0.0009±0.0001 | n.d. | n.d. | n.d. | 0.0022±0 | n.d. | 0.0017±0.0006 | n.d. | n.d. |
| Hexanoic acid, 2-methylpropyl ester | 105-79-3 | 1149 | 1353 | 0.0046±0.0004 | 0.0192±0.0019 | 0.004±0.0002 | 0.0055±0.0003 | 0.0075±0 | n.d. | 0.0316±0.0036 | 0.0078±0.0003 | 0.0117±0.0003 |
| Propanoic acid, 2-hydroxy-, ethyl ester, (L)- | 687-47-8 | 848 | 1359 | 0.0115±0.0004 | 0.0361±0.0012 | 0.0341±0.0002 | 0.0274±0.0007 | 0.0315±0.0003 | n.d. | 0.0215±0.0007 | 0.0353±0.0006 | 0.0342±0.0001 |
| Hexanoic acid, butyl ester | 626-82-4 | 1189 | 1414 | 0.033±0.0035 | 0.0671±0.0004 | 0.0685±0.0025 | 0.0745±0.002 | 0.3115±0.0042 | 0.0549±0.0037 | 0.3196±0.0272 | 0.0315±0.0014 | 0.1178±0.0054 |
| Butanoic acid, hexyl ester | 2639-63-6 | 1192 | 1417 | 0.0098±0.0009 | n.d. | 0.0085±0.0006 | 0.0164±0.0017 | 0.0538±0.0025 | 0.0068±0.0003 | 0.0496±0.0004 | n.d. | 0.0169±0.0006 |
| Cyclohexanecarboxylic acid, ethyl ester | 3289-28-9 | 1136 | 1422 | 0.0011±0.0001 | n.d. | n.d. | n.d. | n.d. | n.d. | n.d. | n.d. | n.d. |
| Octanoic acid, ethyl ester | 106-32-1 | 1196 | 1440 | 0.3079±0.0171 | n.d. | 0.153±0.006 | 0.4357±0.03 | 0.3224±0.0161 | 0.2394±0.0203 | 0.4728±0.0442 | 0.3229±0.0036 | 0.2749±0.042 |
| Isopentyl hexanoate | 2198-61-0 | 1250 | 1460 | 0.0306±0.0004 | 0.0973±0.0042 | 0.0155±0.0012 | 0.0411±0.0075 | 0.0379±0.0025 | 0.0561±0.0009 | 0.2485±0.0128 | 0.054±0.0003 | 0.0828±0.0009 |
| Methyl formate | 107-31-3 | 372 | 1475 | 0.0435±0.0008 | 0.0668±0.0049 | 0.0776±0.0033 | n.d. | 0.0737±0.0004 | 0.0575±0.0008 | 0.0844±0.0063 | 0.0468±0.0011 | 0.0552±0.0016 |
| Hexanoic acid, pentyl ester | 540-07-8 | 1287 | 1513 | 0.0101±0.0003 | 0.0181±0.0047 | 0.0072±0.0012 | 0.0053±0.0003 | 0.03±0.0016 | 0.0074±0 | 0.1691±0.0062 | 0.0049±0.0001 | 0.0232±0.0039 |
| Nonanoic acid, ethyl ester | 123-29-5 | 1295 | 1538 | 0.0057±0.0001 | 0.1168±0.0075 | 0.0052±0.0002 | 0.0118±0.001 | 0.0035±0.0003 | 0.0075±0.0004 | 0.0665±0.0196 | 0.0074±0.0004 | 0.0454±0.0022 |
| Propanoic acid, 2-hydroxy-, pentyl ester | 1637174 | 1075 | 1581 | 0.0006±0 | 0.0038±0 | 0.001±0 | 0.0022±0.0001 | n.d. | 0.0033±0.0001 | n.d. | 0.0051±0.0001 | n.d. |
| Hexanoic acid, hexyl ester | 6378-65-0 | 1384 | 1610 | 0.0548±0.001 | 0.2148±0.0301 | 0.0354±0.0009 | 0.066±0.0042 | 0.068±0.003 | 0.072±0.002 | 0.3696±0.0249 | 0.0365±0.0048 | 0.2688±0.0263 |
| Decanoic acid, ethyl ester | 110-38-3 | 1396 | 1636 | 0.0051±0 | 0.1228±0.0011 | 0.0024±0.0001 | 0.0163±0.0029 | 0.011±0.001 | 0.0156±0 | 0.1687±0.0186 | 0.0168±0.0015 | 0.0956±0.0101 |
| Butanedioic acid, diethyl ester | 123-25-1 | 1181 | 1685 | 0.0032±0 | 0.0261±0.0012 | 0.0077±0.0001 | 0.0192±0.0003 | 0.0077±0.0004 | 0.0406±0.0012 | 0.0103±0.0005 | 0.08±0.003 | 0.037±0.0002 |
| n-Heptyl hexanoate | 6976-72-3 | 1482 | 1717 | 0.0046±0.0002 | n.d. | n.d. | 0.0051±0.0001 | n.d. | n.d. | 0.1751±0.0245 | 0.0016±0.0001 | 0.0123±0.0029 |
| Ethyl trans-2-decenoate | 7367-88-6 | 1389 | 1770 | 0.001±0.0001 | n.d. | n.d. | n.d. | n.d. | n.d. | n.d. | n.d. | n.d. |
| Benzoic acid, 2-hydroxy-, ethyl ester | 118-61-6 | 1270 | 1815 | 0.0002±0 | 0.0001±0 | n.d. | n.d. | n.d. | n.d. | n.d. | n.d. | n.d. |
| Octanoic acid, hexyl ester | 1117-55-1 | 1581 | 1819 | 0.0044±0.0005 | 0.0887±0.0014 | n.d. | 0.0107±0.0023 | 0.0071±0.0017 | 0.0026±0.0003 | 0.1236±0.0167 | 0.003±0 | 0.0356±0.0006 |
| Dodecanoic acid, ethyl ester | 106-33-2 | 1594 | 1861 | 0.0058±0.0004 | 0.0239±0.0011 | 0.0028±0.0002 | 0.0083±0.0008 | 0.0051±0 | 0.0063±0.0001 | n.d. | 0.0082±0 | 0.0184±0.0012 |
| Hexanoic acid, methyl ester | 106-70-7 | 925 | 1921 | 0.005±0.0001 | n.d. | 0.0085±0.0002 | 0.0057±0.0001 | 0.0052±0.0001 | 0.0047±0.0001 | n.d. | 0.0024±0.0002 | n.d. |
| Ethyl tridecanoate | 28267-29-0 | 1687 | 1953 | 0.0012±0 | 0.0028±0.0001 | n.d. | 0.0018±0.0001 | n.d. | n.d. | 0.0051±0.0004 | 0.0014±0.0002 | 0.0032±0.0001 |
| 2(3H)-Furanone, dihydro-5-pentyl- | 104-61-0 | 1365 | 2047 | 0.0022±0.0001 | 0.0088±0.0001 | 0.0041±0.0003 | 0.0061±0.0001 | 0.004±0.0003 | 0.0052±0.0001 | n.d. | 0.0046±0.0003 | 0.0078±0.0004 |
| Tetradecanoic acid, ethyl ester | 124-06-1 | 1793 | 2057 | 0.0347±0.003 | 0.0019±0.0002 | 0.0141±0.0006 | 0.0335±0.0047 | 0.0232±0.0004 | 0.0145±0.0012 | 0.0722±0.0043 | 0.0014±0.0001 | 0.0566±0.0005 |
| Pentadecanoic acid, ethyl ester | 41114-00-5 | 1894 | 2154 | 0.0084±0.0005 | 0.0067±0.0003 | 0.003±0.0005 | 0.0131±0.0023 | 0.0042±0.0003 | n.d. | 0.0231±0.0012 | 0.0048±0.0003 | 0.0129±0.0027 |
| Hexadecanoic acid, ethyl ester | 628-97-7 | 1993 | 2245 | 0.2646±0.0067 | 0.1984±0.1702 | 0.1951±0.0025 | 0.2464±0.0374 | 0.2065±0.0045 | 0.0658±0.0028 | 0.3473±0.009 | 0.1856±0.0243 | 0.3758±0.0354 |
| Ethyl 9-hexadecenoate | 54546-22-4 | 1976 | 2266 | 0.0362±0.0056 | 0.0025±0.0002 | 0.0059±0.0064 | 0.0133±0.0015 | 0.0159±0.0038 | 0.0046±0.0002 | 0.0237±0.0046 | 0.0093±0.0003 | 0.0162±0.0008 |
| Heptadecanoic acid, ethyl ester | 14010-23-2 | 2092 | 2324 | 0.0018±0.0001 | n.d. | n.d. | n.d. | n.d. | n.d. | n.d. | n.d. | n.d. |
| Octadecanoic acid, ethyl ester | 111-61-5 | 2195 | 2401 | 0.0143±0.0027 | 0.0034±0.0002 | 0.007±0.0004 | 0.0065±0.0007 | 0.0093±0.0027 | n.d. | n.d. | 0.0078±0.0003 | 0.0091±0.0021 |
| Ethyl Oleate | 111-62-6 | 2168 | 2418 | 0.1531±0.0343 | 0.062±0.0006 | 0.094±0.007 | 0.0698±0.008 | 0.11±0.0112 | 0.0203±0.0151 | 0.1202±0.0105 | 0.0821±0.003 | 0.1187±0.0234 |
| Linoleic acid ethyl ester | 544-35-4 | 2160 | 2455 | 0.0659±0.0498 | 0.0672±0.0206 | 0.0516±0.0028 | 0.034±0.0046 | 0.0491±0.0018 | 0.0223±0.0161 | 0.0256±0.0291 | 0.0357±0.0008 | 0.0893±0.0126 |
| Propanoic acid, 2-methyl-, propyl ester | 644-49-5 | 820 | 1215 | n.d. | 0.0031±0.0001 | n.d. | n.d. | n.d. | n.d. | 0.006±0.0005 | n.d. | 0.0005±0 |
| Acetic acid, octyl ester | 112-14-1 | 1210 | 1479 | n.d. | 0.0027±0.0002 | n.d. | n.d. | n.d. | n.d. | 0.003±0.0001 | n.d. | n.d. |
| Propyl octanoate | 624-13-5 | 1290 | 1522 | n.d. | 0.0223±0.0015 | n.d. | n.d. | 0.0024±0.0001 | n.d. | 0.0298±0.0001 | n.d. | 0.0065±0 |
| Pentanoic acid, 2-hydroxy-4-methyl-, ethyl ester | 10348-47-7 | 1060 | 1556 | n.d. | 0.0437±0.0004 | 0.0245±0.0003 | 0.0082±0.0005 | 0.0212±0.0007 | 0.0181±0.0004 | n.d. | 0.0243±0.0001 | 0.0156±0.0002 |
| Heptanoic acid, 3-methylbutyl ester | 109-25-1 | 1347 | 1560 | n.d. | 0.0114±0.0013 | n.d. | n.d. | n.d. | 0.001±0.0001 | 0.0223±0.0013 | 0.001±0 | n.d. |
| Propanoic acid, 2-methyl-, octyl ester | 109-15-9 | 1344 | 1617 | n.d. | 0.0057±0.0004 | n.d. | n.d. | n.d. | n.d. | n.d. | n.d. | n.d. |
| Octanoic acid, 3-methylbutyl ester | 2035-99-6 | 1446 | 1658 | n.d. | 0.0631±0.0042 | n.d. | 0.0017±0.0004 | n.d. | n.d. | 0.0235±0.0016 | 0.0013±0.0001 | 0.0066±0.0004 |
| Undecanoic acid, ethyl ester | 627-90-7 | 1494 | 1751 | n.d. | 0.0053±0.0003 | n.d. | n.d. | n.d. | n.d. | 0.0102±0.0033 | n.d. | n.d. |
| Butanoic acid, pentyl ester | 540-18-1 | 1077 | 1118 | n.d. | n.d. | 0.0016±0.0002 | n.d. | n.d. | n.d. | n.d. | n.d. | n.d. |
| Acetic acid, pentyl ester | 628-63-7 | 910 | 1171 | n.d. | n.d. | 0.0007±0.0001 | n.d. | n.d. | n.d. | n.d. | n.d. | n.d. |
| Butanoic acid, butyl ester | 109-21-7 | 995 | 1217 | n.d. | n.d. | 0.0082±0.0001 | 0.0088±0.0007 | 0.1062±0.0057 | n.d. | n.d. | n.d. | 0.0052±0.0001 |
| Methyl salicylate | 119-36-8 | 1192 | 1782 | n.d. | n.d. | 0.002±0 | n.d. | n.d. | 0.0032±0.0002 | n.d. | n.d. | n.d. |
| n-Propyl acetate | 109-60-4 | 712 | 1876 | n.d. | n.d. | 0.025±0.0052 | n.d. | n.d. | n.d. | n.d. | n.d. | n.d. |
| 1-Butanol, 3-methyl-, acetate | 123-92-2 | 876 | 1119 | n.d. | n.d. | n.d. | 0.0015±0.0001 | n.d. | 0.0075±0.0005 | n.d. | 0.0069±0.0001 | n.d. |
| Diethyl azelate | 624-17-9 | 1688 | 2214 | n.d. | n.d. | n.d. | 0.0077±0.0002 | n.d. | n.d. | n.d. | n.d. | n.d. |
| Butanoic acid, 2-methylpropyl ester | 539-90-2 | 954 | 1151 | n.d. | n.d. | n.d. | n.d. | 0.001±0.0001 | n.d. | n.d. | n.d. | n.d. |
| Butanoic acid, 3-methyl-, butyl ester | 109-19-3 | 1045 | 1249 | n.d. | n.d. | n.d. | n.d. | 0.0037±0.0006 | n.d. | n.d. | n.d. | n.d. |
| Isobutyl isovalerate | 589-59-3 | 1005 | 1255 | n.d. | n.d. | n.d. | n.d. | 0.0004±0 | n.d. | 0.0007±0 | 0.0003±0 | n.d. |
| Butanoic acid, 3-methyl-, 3-methylbutyl ester | 659-70-1 | 1104 | 1362 | n.d. | n.d. | n.d. | n.d. | 0.0023±0.0001 | n.d. | n.d. | n.d. | n.d. |
| Heptanoic acid, propyl ester | 7778-87-2 | 1183 | 1420 | n.d. | n.d. | n.d. | n.d. | 0.0025±0.0002 | n.d. | 0.0124±0.0016 | n.d. | n.d. |
| Butanoic acid, 3-methyl-, hexyl ester | 10032-13-0 | 1244 | 1447 | n.d. | n.d. | n.d. | n.d. | 0.0007±0 | n.d. | n.d. | n.d. | n.d. |
| Butyl caprylate | 589-75-3 | 1389 | 1616 | n.d. | n.d. | n.d. | n.d. | 0.0037±0.0003 | n.d. | n.d. | n.d. | n.d. |
| Butanedioic acid, ethyl 3-methylbutyl ester | 28024-16-0 | 1436 | 1913 | n.d. | n.d. | n.d. | n.d. | n.d. | n.d. | n.d. | 0.0015±0.0001 | n.d. |
| **Aldehydes** |  |  |  |  |  |  |  |  |  |  |  |  |
| Nonanal | 124-19-6 | 1104 | 1395 | n.d. | 0.0111±0.005 | n.d. | n.d. | n.d. | 0.0249±0.0185 | 0.0046±0.0004 | 0.0018±0.0001 | 0.0092±0.0001 |
| **Ketones** |  |  |  |  |  |  |  |  |  |  |  |  |
| 2-Octanone | 111-13-7 | 991 | 1286 | 0.0023±0.0001 | 0.0013±0.0001 | 0.0022±0.0016 | 0.002±0 | 0.0018±0.0002 | 0.0016±0.0005 | 0.0014±0.0002 | 0.0016±0.0001 | n.d. |
| 2-Nonanone | 821-55-6 | 1092 | 1391 | 0.0018±0.0001 | n.d. | n.d. | n.d. | n.d. | n.d. | n.d. | n.d. | n.d. |
| 2-Pentadecanone | 2345-28-0 | 1698 | 2028 | 0.0029±0.0001 | n.d. | n.d. | n.d. | n.d. | n.d. | n.d. | n.d. | n.d. |
| 2H-Pyran-2-one, 6-pentyl- | 27593-23-3 | 1453 | 2192 | 0.0003±0 | n.d. | n.d. | n.d. | n.d. | n.d. | n.d. | n.d. | n.d. |
| Propanoic acid, 2-oxo-, methyl ester | 600-22-6 | 701 | 2212 | 0.0061±0.0043 | n.d. | n.d. | n.d. | n.d. | 0.006±0 | n.d. | n.d. | n.d. |
| **Aromatic and phenolic compounds** |  |  |  |  |  |  |  |  |  |  |  |  |
| Toluene | 108-88-3 | 763 | 1047 | 0.0022±0.0029 | n.d. | n.d. | n.d. | 0.004±0.0004 | n.d. | n.d. | n.d. | n.d. |
| Styrene | 100-42-5 | 893 | 1258 | 0.0014±0.0002 | 0.0007±0.0001 | 0.001±0.0002 | 0.0059±0.0003 | 0.0027±0.0002 | 0.0008±0 | n.d. | 0.0013±0.0001 | 0.0012±0 |
| Benzaldehyde | 100-52-7 | 962 | 1530 | 0.017±0.0007 | 0.0616±0.0032 | 0.0304±0.0011 | 0.0386±0.0011 | 0.0506±0.0006 | 0.0408±0.0012 | 0.0566±0.0005 | 0.0408±0.0025 | 0.0286±0.0009 |
| Acetophenone | 98-86-2 | 1066 | 1652 | 0.0005±0.0001 | 0.001±0.0002 | 0.0007±0 | 0.0007±0 | 0.001±0 | 0.0009±0.0001 | 0.0084±0.0005 | n.d. | 0.0004±0 |
| Benzoic acid, ethyl ester | 93-89-0 | 1172 | 1668 | 0.0264±0.0005 | 0.0026±0.0001 | 0.003±0.0001 | 0.0066±0.0003 | 0.0028±0.0001 | 0.0062±0.0002 | 0.0039±0.0001 | 0.0042±0.0003 | n.d. |
| Benzeneacetic acid, ethyl ester | 101-97-3 | 1247 | 1795 | 0.038±0.0004 | 0.0717±0.0026 | n.d. | 0.052±0.0019 | 0.0293±0.0012 | 0.0724±0.0032 | 0.0415±0.0014 | 0.0329±0.0006 | 0.0388±0.0011 |
| Acetic acid, 2-phenylethyl ester | 103-45-7 | 1258 | 1825 | 0.0054±0.0001 | 0.0134±0.0003 | n.d. | 0.0092±0.0001 | 0.006±0.0001 | 0.0095±0.0002 | n.d. | 0.0074±0.0003 | n.d. |
| Phenylacetic acid propyl ester | 4606-15-9 | 1331 | 1882 | 0.0002±0 | 0.0003±0 | 0.0006±0 | n.d. | 0.0003±0 | 0.0002±0 | n.d. | n.d. | n.d. |
| Benzenepropanoic acid, ethyl ester | 2021-28-5 | 1353 | 1895 | 0.0736±0.0017 | 0.1867±0.0022 | 0.0801±0.0026 | 0.1144±0.0206 | 0.1167±0.0063 | 0.0711±0.0047 | 0.2608±0.0238 | 0.099±0.0042 | 0.1072±0.0036 |
| Phenylethyl Alcohol | 22258 | 1116 | 1930 | 0.062±0.0007 | 0.1207±0.0034 | 0.0582±0.0004 | 0.0875±0.0034 | 0.0659±0.0029 | 0.0801±0.0018 | 0.0612±0.0027 | 0.0806±0.0006 | 0.0858±0.0015 |
| Propanoic acid, 2-methyl-, 2-phenylethyl ester | 103-48-0 | 1396 | 1975 | 0.0016±0.0001 | n.d. | n.d. | n.d. | n.d. | 0.0008±0 | n.d. | n.d. | n.d. |
| 2-Methoxy-5-methylphenol | 1195-09-1 | 1193 | 1977 | 0.0025±0.0001 | n.d. | 0.0039±0.0002 | 0.0218±0.0034 | 0.0246±0.001 | 0.0185±0 | 0.0152±0.002 | 0.0396±0.0016 | 0.032±0.0011 |
| Phenol | 108-95-2 | 981 | 2037 | 0.007±0.0002 | 0.0241±0.0008 | 0.0246±0.0003 | 0.0192±0.0007 | 0.0051±0.0001 | 0.023±0.0006 | 0.0239±0.002 | 0.0131±0.0004 | 0.0245±0.0012 |
| Phenol, 4-ethyl-2-methoxy- | 2785-89-9 | 1282 | 2053 | 0.0132±0.0003 | 0.0158±0.0012 | 0.0022±0.0001 | 0.0123±0.0002 | 0.0064±0.0004 | 0.0118±0.0003 | 0.0202±0.0026 | 0.0744±0.0037 | 0.0564±0.0023 |
| p-Cresol | 106-44-5 | 1077 | 2112 | 0.0973±0.0008 | 0.0472±0.0814 | 0.0003±0 | 0.1191±0.0103 | 0.0249±0.0015 | 0.0968±0.0011 | 0.1555±0.0004 | 0.1127±0.0046 | 0.1028±0.0034 |
| Isophthalaldehyde | 626-19-7 | 1284 | 2154 | 0.0002±0.0001 | 0.0012±0.0003 | n.d. | n.d. | n.d. | 0.0017±0.0002 | n.d. | n.d. | n.d. |
| Hexanoic acid, 2-phenylethyl ester | 6290-37-5 | 1650 | 2178 | 0.0084±0.0006 | 0.0304±0.0022 | 0.0101±0.0005 | 0.018±0.0005 | 0.0092±0.001 | n.d. | 0.0635±0.0034 | n.d. | 0.0182±0.0008 |
| Phenol, 4-ethyl- | 123-07-9 | 1169 | 2197 | 0.0033±0.0001 | 0.0149±0.0004 | 0.0005±0 | 0.0058±0.0001 | 0.0027±0.0002 | 0.0101±0.0001 | 0.0083±0 | 0.0178±0.0006 | 0.0165±0.0005 |
| Ethyl 2-hydroxy-3-phenylpropanoate | 15399-05-0 | 1432 | 2279 | 0.0094±0.0002 | 0.1425±0.0059 | 0.0228±0.0009 | 0.0287±0.0002 | 0.0458±0.0011 | 0.0524±0.0046 | 0.0467±0.001 | 0.0889±0.0024 | 0.0866±0.0025 |
| 2,4-Di-tert-butylphenol | 96-76-4 | 1514 | 2302 | 0.0062±0.0002 | 0.0048±0.0001 | 0.0058±0.0002 | 0.0052±0 | 0.0054±0.0003 | 0.0056±0.0001 | n.d. | 0.0056±0.0001 | 0.0051±0.0003 |
| Benzophenone | 119-61-9 | 1635 | 2437 | 0.0003±0.0001 | 0.0009±0.0002 | n.d. | 0.0002±0 | n.d. | 0.0017±0.0001 | n.d. | 0.0001±0 | n.d. |
| Benzeneacetic acid | 103-82-2 | 1262 | 2506 | 0.0089±0.0008 | 0.0075±0.0124 | 0.0331±0.0005 | 0.0085±0.0002 | 0.0052±0.0001 | 0.0233±0.0044 | 0.0271±0.004 | 0.0109±0.0022 | 0.014±0.0007 |
| Naphthalene | 91-20-3 | 1182 | 1745 | n.d. | 0.0016±0.0001 | 0.0014±0.0002 | 0.0017±0 | n.d. | 0.0028±0.0002 | n.d. | 0.0017±0.0001 | n.d. |
| Naphthalene, 2-methyl- | 91-57-6 | 1297 | 1854 | n.d. | 0.0004±0 | 0.0001±0.0001 | n.d. | n.d. | 0.0006±0 | n.d. | 0.0002±0 | n.d. |
| Benzenepropanoic acid 1-methylethyl ester | 22767-95-9 | 1385 | 1984 | n.d. | 0.0013±0.0001 | n.d. | 0.0003±0 | 0.0011±0.0001 | n.d. | 0.0046±0.0001 | n.d. | 0.0004±0 |
| Biphenyl | 92-52-4 | 1381 | 1998 | n.d. | 0.0004±0 | n.d. | n.d. | n.d. | n.d. | n.d. | n.d. | n.d. |
| 4-Vinylbenzoic acid | 1075-49-6 | 1353 | 2712 | n.d. | 0.0013±0.0004 | n.d. | n.d. | n.d. | 0.0026±0.0001 | n.d. | n.d. | n.d. |
| Benzeneacetaldehyde | 122-78-1 | 1045 | 1648 | n.d. | n.d. | 0.0035±0.0004 | 0.0037±0.0001 | 0.0028±0 | n.d. | 0.0056±0.0002 | 0.0032±0 | n.d. |
| Benzeneacetic acid, butyl ester | 122-43-0 | 1440 | 1973 | n.d. | n.d. | 0.0017±0.0001 | n.d. | 0.0013±0.0001 | n.d. | n.d. | n.d. | n.d. |
| Naphthalene, 2-ethenyl- | 827-54-3 | 1407 | 1999 | n.d. | n.d. | 0.0003±0 | n.d. | n.d. | n.d. | n.d. | n.d. | n.d. |
| Butanoic acid, 3-methyl-, 2-phenylethyl ester | 140-26-1 | 1491 | 2080 | n.d. | n.d. | 0.0008±0 | n.d. | n.d. | n.d. | n.d. | 0.0006±0.0001 | n.d. |
| Phenol, 3,5-dimethyl- | 108-68-9 | 1171 | 1252 | n.d. | n.d. | n.d. | 0.0002±0 | n.d. | n.d. | n.d. | n.d. | n.d. |
| Butanedioic acid, phenyl- | 635-51-8 | 1772 | 1265 | n.d. | n.d. | n.d. | 0.0004±0 | n.d. | n.d. | 0.0015±0.0001 | n.d. | n.d. |
| n-Propyl benzoate | 2315-68-6 | 1286 | 1882 | n.d. | n.d. | n.d. | 0.0001±0 | n.d. | n.d. | n.d. | n.d. | n.d. |
| .beta.-Phenylethyl butyrate | 103-52-6 | 1444 | 1976 | n.d. | n.d. | n.d. | 0.0034±0.0002 | n.d. | n.d. | n.d. | n.d. | n.d. |
| Mesitylene | 108-67-8 | 972 | 1280 | n.d. | n.d. | n.d. | n.d. | n.d. | n.d. | n.d. | 0.0001±0 | 0.0001±0 |
| Ethanone, 1-(1H-pyrrol-2-yl)- | 1072-83-9 | 1063 | 1995 | 0.0003±0 | n.d. | 0.0008±0 | 0.0003±0 | 0.0003±0 | 0.0004±0 | 0.0016±0.0002 | n.d. | 0.0006±0 |
| 1H-Indole, 4-methyl- | 16096-32-5 | 1288 | 2451 | n.d. | 0.0008±0 | n.d. | n.d. | n.d. | 0.0008±0.0001 | n.d. | 0.0005±0 | 0.0008±0.0001 |
| **Furan derivatives** |  |  |  |  |  |  |  |  |  |  |  |  |
| Furfural | 35796 | 833 | 1478 | 0.0017±0 | 0.0047±0.0003 | 0.0134±0.0001 | 0.0024±0.0001 | 0.0018±0.0001 | 0.0022±0 | 0.0051±0.0001 | 0.0049±0 | n.d. |
| 1-Propanone, 1-(2-furanyl)- | 3194-15-8 | 1012 | 1586 | 0.0004±0 | 0.0005±0 | 0.0004±0 | 0.0003±0 | 0.0007±0 | 0.0005±0 | 0.0008±0 | 0.0006±0 | n.d. |
| 2-Acetyl-5-methylfuran | 1193-79-9 | 1039 | 1621 | 0.0006±0 | 0.0004±0.0001 | 0.0007±0 | n.d. | 0.0003±0 | 0.0001±0 | n.d. | 0.0001±0 | 0.0002±0 |
| 2-Furanmethanol | 98-00-0 | 860 | 1677 | 0.0073±0.0002 | 0.0082±0.0007 | 0.0124±0.0002 | 0.0078±0.0004 | 0.0054±0.0004 | 0.0067±0.0001 | 0.0144±0.0007 | n.d. | 0.0042±0.0001 |
| Furfuryl hexanoate | 39252-02-3 | 1368 | 1881 | n.d. | 0.002±0.0004 | n.d. | 0.0025±0.0001 | n.d. | n.d. | 0.0124±0.0021 | n.d. | 0.0016±0.0001 |
| 2-Furanmethanol, acetate | 623-17-6 | 995 | 1550 | n.d. | n.d. | 0.0004±0 | 0.0002±0 | n.d. | 0.0002±0 | 0.0006±0.0001 | n.d. | n.d. |
| **Pyrazines** |  |  |  |  |  |  |  |  |  |  |  |  |
| Pyrazine, tetramethyl- | 1124-11-4 | 1088 | 1478 | 0.0025±0.0002 | 0.0008±0.0001 | 0.0009±0 | n.d. | 0.0011±0.0001 | n.d. | 0.0005±0 | 0.0012±0 | n.d. |
| Pyrazine, 2,6-dimethyl- | 108-50-9 | 917 | 1333 | n.d. | 0.0006±0 | n.d. | n.d. | n.d. | 0.0002±0 | n.d. | n.d. | n.d. |
| Pyrazine, trimethyl- | 14667-55-1 | 1004 | 1407 | n.d. | n.d. | n.d. | n.d. | 0.0002±0 | n.d. | n.d. | n.d. | n.d. |
| Pyrazine, 3-ethyl-2,5-dimethyl- | 13360-65-1 | 1081 | 1449 | n.d. | n.d. | n.d. | n.d. | 0.0001±0 | n.d. | n.d. | n.d. | n.d. |
| Sulfur-containing compounds |  |  |  |  |  |  |  |  |  |  |  |  |
| 3-(Methylthio)propanoic acid ethyl ester | 13327-56-5 | 1098 | 1575 | 0.0005±0 | 0.001±0.0001 | 0.0013±0 | 0.0007±0 | 0.0007±0 | 0.0014±0 | n.d. | 0.0011±0 | 0.0006±0 |
| 2-Methylthioacetic acid | 2444-37-3 | 927 | 1736 | 0.0005±0.0003 | n.d. | 0.0003±0.0002 | n.d. | n.d. | n.d. | n.d. | n.d. | n.d. |
| Butanethioic acid, S-methyl ester | 2432-51-1 | 888 | 1194 | n.d. | 0.0005±0 | 0.0007±0 | 0.0005±0 | n.d. | 0.0006±0 | n.d. | 0.0007±0 | 0.0004±0 |
| Dimethyl trisulfide | 3658-80-8 | 971 | 1382 | n.d. | n.d. | n.d. | n.d. | n.d. | n.d. | n.d. | 0.0001±0 | n.d. |
| **Other categories** |  |  |  |  |  |  |  |  |  |  |  |  |
| 1,3-Cyclopentadiene, 1,2,3,4,5-pentamethyl- | 4045-44-7 | 983 | 1275 | 0.0002±0 | n.d. | n.d. | n.d. | n.d. | n.d. | n.d. | n.d. | n.d. |
| Tridecane | 629-50-5 | 1300 | 1289 | 0.0048±0.0005 | 0.004±0.0002 | 0.0053±0.0001 | n.d. | 0.0054±0.0001 | 0.0075±0.0013 | 0.0033±0.0003 | 0.0048±0.0004 | 0.0053±0.0002 |
| Ammonium acetate | 631-61-8 | 630 | 1693 | 0.045±0.0318 | 0.0153±0.0008 | n.d. | 0.0588±0.0028 | n.d. | n.d. | 0.015±0.0015 | n.d. | 0.0081±0 |
| Heptadecane | 629-78-7 | 1700 | 1700 | 0.0016±0.0001 | 0.0027±0.0002 | 0.0016±0.0002 | 0.0011±0.0001 | 0.0036±0.0003 | 0.0051±0 | n.d. | 0.0024±0.0001 | 0.0011±0 |
| Octadecane | 593-45-3 | 1800 | 1803 | 0.0016±0.0002 | 0.0026±0.0002 | n.d. | n.d. | n.d. | 0.0042±0.0007 | n.d. | 0.0016±0.0004 | n.d. |
| Hexadecane | 544-76-3 | 1600 | 1600 | n.d. | 0.002±0.0014 | n.d. | n.d. | n.d. | 0.0041±0.0002 | n.d. | n.d. | n.d. |
| Hexanoic acid, anhydride | 2051-49-2 | 1517 | 1960 | n.d. | n.d. | 0.0031±0.0002 | n.d. | n.d. | n.d. | 0.0436±0.0013 | n.d. | n.d. |
| 1,3-Cyclohexadiene, 1-methyl-4-(1-methylethyl)- | 99-86-5 | 1017 | 1275 | n.d. | n.d. | n.d. | n.d. | 0.0015±0.0001 | n.d. | n.d. | n.d. | n.d. |

* The mean ± SD of measurements made in triplicates were used to reflect the characteristic values. MW: molecular weight; RI: retention index; n.d.: volatile compounds not detected.
